# Are the gut microbial systems of giant pandas unstable?

**DOI:** 10.1016/j.heliyon.2019.e02480

**Published:** 2019-09-17

**Authors:** Ran Yao, Zhisong Yang, Zheng Zhang, Ting Hu, Hua Chen, Feng Huang, Xiaodong Gu, Xuyu Yang, Guoqing Lu, Lifeng Zhu

**Affiliations:** a College of Life Sciences, Nanjing Normal University, Nanjing, 210023, China; b Key Laboratory of Southwest China Wildlife Resources Conservation (Ministry of Education), China West Normal University, Nanchong, 637002, China; c University of Nebraska at Omaha, Omaha, USA; d Sichuan Lizhiping Giant Panda National Nature Reserve, Shimian, China; e Sichuan Station of Wild Life Survey and Management, Chengdu, 610082, China; f Shanghai Biozeron Bioinfmatics Center, Shanghai, 201800, China

**Keywords:** Microbiology, Adaptation, Population dynamics, Bacteria, Genome sequencing, High-throughput sequencing, Coevolution, Bamboo, Giant pandas, Red pandas, Gut microbial system

## Abstract

Animals have stable dominant gut microbiomes under similar diets. Similar diets can also lead to similar gut microbial communities within host species levels. Giant pandas (*Ailuropoda melanoleuca*) and red pandas (*Ailurus fulgens*) have had long-term and stable bamboo diets, and seem well adapted to this highly fibrous diet. When compared to the gut microbiomes of Père David's deer (*Elaphurus davidianus*), humans, cheetah (*Acinonyx jubatus*), black-backed jackal (*Canis-mesomelas*), and black bear (*Ursus thibetanus*), giant panda gut microbiomes have high variation in the abundance of Pseudomonadaceae and Clostridiaceae, and are somewhat unstable. This high instability and dissimilarity may reflect an unstable gut environment, perturbation or selective pressure because of their carnivorous gastrointestinal system. A short digestive tract, brief digestion time and fast intestinal peristalsis may result in higher oxygen concentrations that select for the growth of aerobes and facultative anaerobes in giant pandas. Potential selection of high proportion of Pseudomonadaceae in giant panda (GP-HP) and red panda gut microbiomes may arise because of their postulated ability to degrade secondary compounds (e.g., cyanide compounds and aromatic compounds). However, high proportion of Clostridiaceae (GP-HF) may focus on cellulose and hemicellulose digestion. Thus, GP-HP and GP-HF groups have high dissimilarity on the functional level. These findings show that long-term similarities in diet do not always lead to similar or stable gut microbial system within the same host species and that other factors can drive the selection of gut taxa.

## 1 Introduction

Typical microbial colonies found on or in the body are normally benign or beneficial (Kau et al., 2011). The mammal gut microbiota protects against enteropathogens, extracts nutrients and energy from diets, and contributes to normal immune function (Sonnenburg et al., 2005; Ley et al., 2008; Fukuda et al., 2011; Pope et al., 2011; Olszak et al., 2012; Yatsunenko et al., 2012). One of the most striking aspects of these complex communities in humans is the long-term stability seen in healthy individuals, whereby the composition of the microbiome shows remarkable permanence (Lozupone et al., 2012; Faith et al., 2013; David et al., 2014; Gerber, 2014; Weingarden et al., 2015; McNally and Brown, 2016; Bian et al., 2017; Gibson et al., 2017). Although temporal dynamics have been found, dominant gut microbial phyla (e.g. Firmicutes) in humans are relative stable (Davenport et al., 2014; Turroni et al., 2017). Stability of a core gut microbiome has also been found in other animals, such as insects, fishes and mammals (Sullam et al., 2015; Tung et al., 2015; Kwong and Moran, 2016; Tinker and Ottesen, 2016; Li et al., 2017; Menke et al., 2017). Ecological and evolutionary forces (host level top-down selection and bottom-up selection) shape microbial diversity and stability in the human intestine (Ley et al., 2006; Moya and Ferrer, 2016). Host diet and phylogeny both influence gut microbiome communities, and within a species such as humans, balance or stability of the core gut microbiome is maintained under the same or similar diet (Benson et al., 2010; Schnorr et al., 2014; Voreades et al., 2014; Hale et al., 2018). The coevolution has been invoked to describe the formation of the host-gut microbe relationship (Amato, 2013). Commonly, this coevolution within the same host is relatively clear: similar diet leads to similar gut microbial communities.

The giant panda (*Ailuropoda melanoleuca*) and red panda (*Ailurus fulgens*) exhibit dietary peculiarities as members of the mammalian order Carnivora because they possess a gastrointestinal tract typical of carnivores yet are bamboo specialists. Giant pandas consume ∼12.5 kg of highly fibrous bamboo material including stems, leaves and shoots each day (Schaller, 1985). Cranial anatomy of the first skull of the earliest giant panda (*A*. *microta*) demonstrates that the specialized cranial and dental adaptations of *Ailuropoda* for durophagous feeding behavior centered on bamboo were already present in the late Pliocene (Jin et al., 2007). Dental remains indicate that the giant panda lineage evolved a precursor stage of crushing dentition by ∼7 Myr ago as seen in *Ailurarctos*, initiating the trend toward a massive, robust skull and jaw for durophagous mastication. The *A. microta* skull indicates that the giant panda could have developed the dependence on bamboo by 2 Myr ago (Jin et al., 2007). Molecular evidence reveals pseudogenization of the umami taste receptor gene Tas1r1 in the giant panda and that functional constraint on giant panda Tas1r1 was relaxed ∼4.2 Myr, coinciding with its dietary switch to bamboo (Zhao et al., 2010). The putative harboring of cellulose- and hemicellulose-digesting microbes in the gut of the giant panda, along with other traits such as pseudothumbs, well-developed teeth, mandible and skull morphology, and chewing muscles, likely arose as a result of adaptation to a highly fibrous bamboo diet (Zhang et al., 2007; Zhu et al., 2011; Wei et al., 2012, 2014). High-volume bamboo ingestion, short digestion time (short food retention time), low digestion of bamboo, short digestive tracts and fast intestinal peristalsis may result in gut perturbation and higher oxygen concentrations that select for the growth of aerobes and facultative anaerobes (Zhang et al., 1995). Dietary fiber interacts with gut epithelium and mucus directly, and may also enhance animal gut perturbation (Montagne et al., 2003). Therefore, the giant panda may be an ideal model to truly explore the co-evolution of host and microbe during their long evolutionary history.

Considering their carnivorous digestive system, our hypothesis was that bamboo is a daily environmental perturbation factor impacting the giant panda gut microbiome and resulting in gut community instability although they have eaten bamboo long time ago. To address this hypothesis, we compared the gut microbiomes of bamboo-eating pandas (giant pandas and red pandas) with the gut microbiomes of Père David's deer (*Elaphurus davidianus*), humans, cheetah (*Acinonyx jubatus*), black-backed jackal (*Canis-mesomelas*), and black bear (*Ursus thibetanus*).

## 2 Results

### 2.1 Gut microbiome composition of pandas

**Fig. 1:**
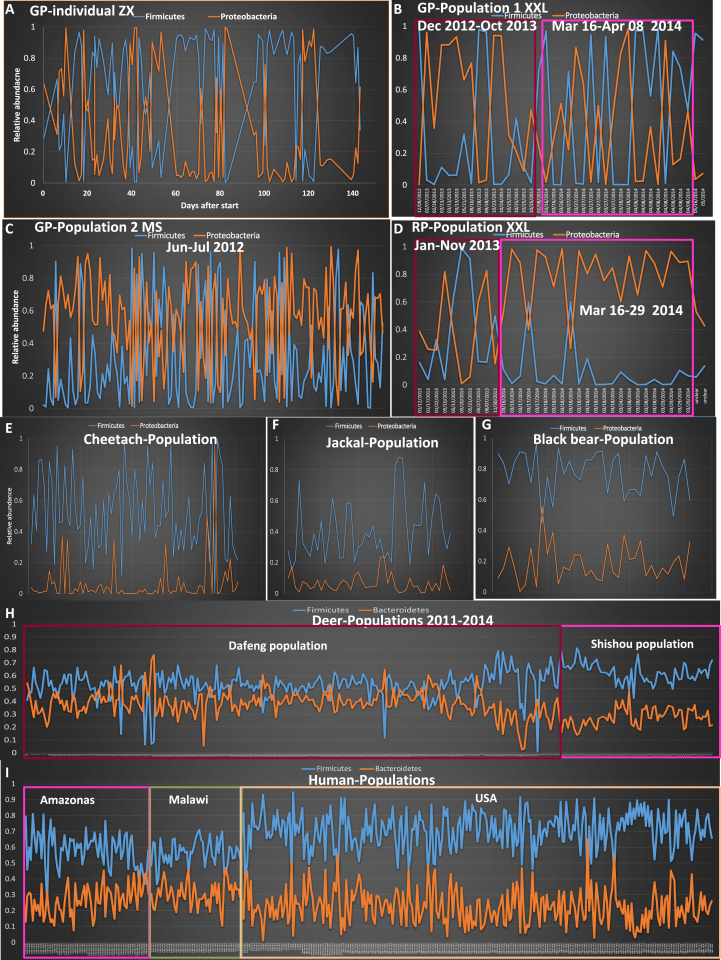
The variation in dominant gut microbial groups (phylum level) among the giant panda and red panda, Père David's deer, cheetah, jackal, black bear, and human. The distribution of dominant gut bacterial phyla in the giant panda individual-Zhangxiang ZX (A), Xiaoxiangling XXL giant panda population (B), Minshan MS giant panda population (C), Xiaoxiangling red panda population (D), Cheetach population (Menke et al., 2014) (E), Jackal population (Menke et al., 2014) (F), black bear population (Song et al., 2017) (G), Père David's deer (H) and humans (Yatsunenko et al., 2012) (I). Y-axis was the relative abundance. X-axis was each sample. Blue line: Firmicutes. Brown line: Proteobacteria.

8,805,818 high-quality 16S ribosomal RNA gene sequences were gained from the feces of giant pandas (n = 318) and red pandas (n = 37). Primary phyla were the Proteobacteria and Firmicutes, and the ratio of these two groups was highly variable (range 0.003–1819) across different samples from the same individual and across populations at the same sampling time (Fig. 1A–D). For instance, in the translocated individual of captive origin (Zhangxiang) sampled in the first five months after translocation, 52 of 102 samples were high Firmicutes and 19 were high Proteobacteria (Fig. 1A). The high variation in the relative abundance of Proteobacteria and Firmicutes were also found in other two captive origin translocated individuals (HJ and TT) (Fig. S1A and S1B). Moreover, this pattern was also observed in the wild Xiaoxiangling giant panda population (Fig. 1B), wild Minshan giant panda population (Fig. 1C), the wild-origin translocated individual (LX) (Fig. S1C) and wild Xiaoxiangling red panda population (19 of 37 samples) (Fig. 1D and Table S1). For example, in wild Minshan giant panda populations, 19 of 139 samples (at the same sampling period) exhibited high Firmicutes abundances (mean >75%). Sixty-two of the 139 Minshan panda samples exhibited high Proteobacteria abundance (mean >75%) and others were median types (M) (Fig. 1C and Table S1).

The most abundant bacterial families detected in wild giant pandas were Pseudomonadaceae (Proteobacteria; Rank 1, 0.2473 ± 0.2772), Clostridiaceae (Clostridiaceae 1, Firmicutes; Rank 2, 0.1955 ± 0.2449) and Enterobacteriaceae (Proteobacteria; Rank 3, 0.0957 ± 0.1772) (Fig. 1B and C). For example, most of the high Proteobacteria types (GP-HP) have the highest abundance of the Pseudomonadaceae family and most of the high Firmicutes types (GP-HF) had the highest abundance of the Clostridiaceae family. The genus *Pseudomonas* comprised the majority of the Pseudomonadaceae from the wild giant and red pandas (0.2473 ± 0.2772 and 0.4537 ± 0.3843, respectively). In contrast, *Clostridium* sensu stricto 1, a genus of gram-positive Firmicutes and obligate anaerobes, comprised the next major group of Clostridiaceae-related bacteria in wild giant panda gut communities (0.1954 ± 0.2450). Therefore, the predominant gut microbiome in the giant panda and red panda were Proteobacteria, Firmicutes, and Bacteroidetes. The mean proportion of Archaea was about 1.6e-5.

### 2.2 Normality testing of major gut microbiomes in pandas, deer and humans

Normality tests are used to determine whether a data set is well-modeled by a normal distribution. Here, we wanted to look at this distribution on the relative abundance of the gut microbiome (dominant phylum level) from different mammal species. We tried to show whether the relative abundance of the dominant microbial phylum is normal distribution (reflecting stable) or randomly distribution. The dominant phylum in the three Carnivora species (Fig. 1E-G), especially in panda's relative (same family: Ursidae)-black bear (although from different habitats), were relatively stable. Moreover, we produced 2,697,940 16S ribosomal RNA gene sequences from 315 deer fecal samples (Table S2) and reanalyzed 16S ribosomal RNA gene sequences from 389 human fecal samples from three human populations (USA, Malawian and Amazonas populations) (Yatsunenko et al., 2012), and also found relatively stable gut microbiomes at the phylum level (Fig. 1H). Firmicutes (∼54%) and Bacteroidetes (∼36%) were the two dominant phyla in the deer gut with very little variation across individuals (Fig. 1I). The highly uniform nature of the human (Kolmogorov-Smirnov test *p* > 0.05 for Firmicutes and Bacteroidetes, respectively) and deer gut microbiome (Kolmogorov-Smirnov test *p* > 0.05 for Bacteroidetes) was exemplified by mostly normally distributed abundances of main core microbiomes compared to those in giant pandas (Kolmogorov-Smirnov test *p* = 0.000 for Proteobacteria and Firmicutes, respectively).

### 2.3 Gut microbiome diversity among pandas, deer, humans, cheetah, black-backed jackal, and black bear

**Fig. 2:**
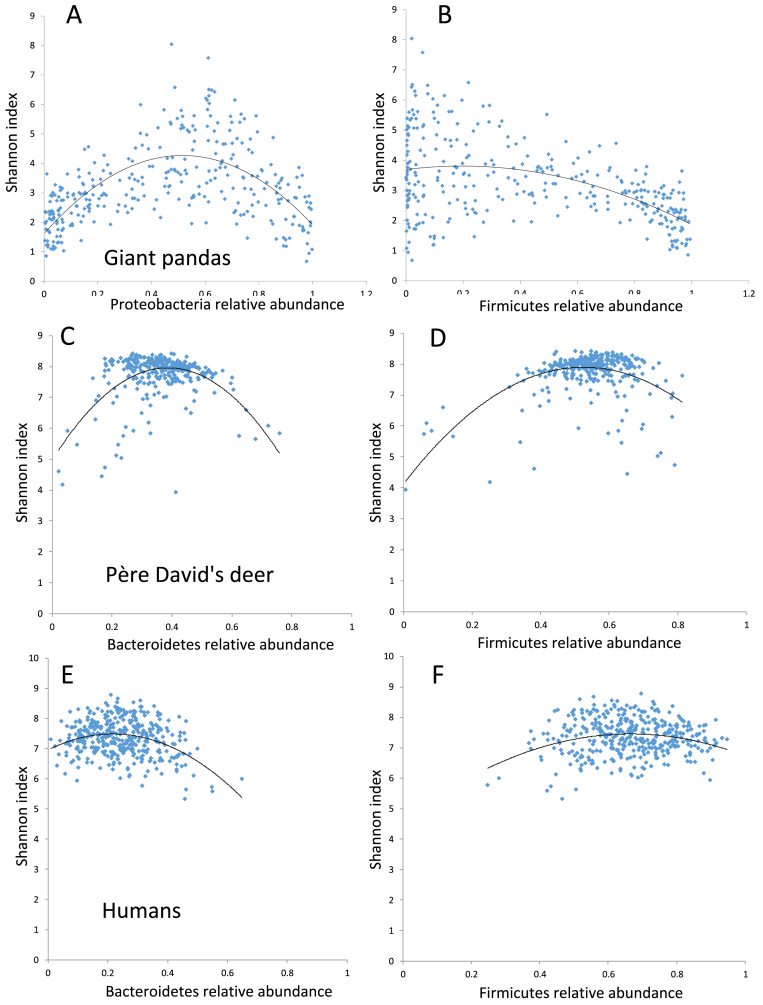
Regression analysis for major gut microbiomes with Shannon diversity index shows a more discrete distribution in giant pandas (A and B) compared with deer or humans (Yatsunenko et al., 2012) (C–F).

**Fig. 3:**
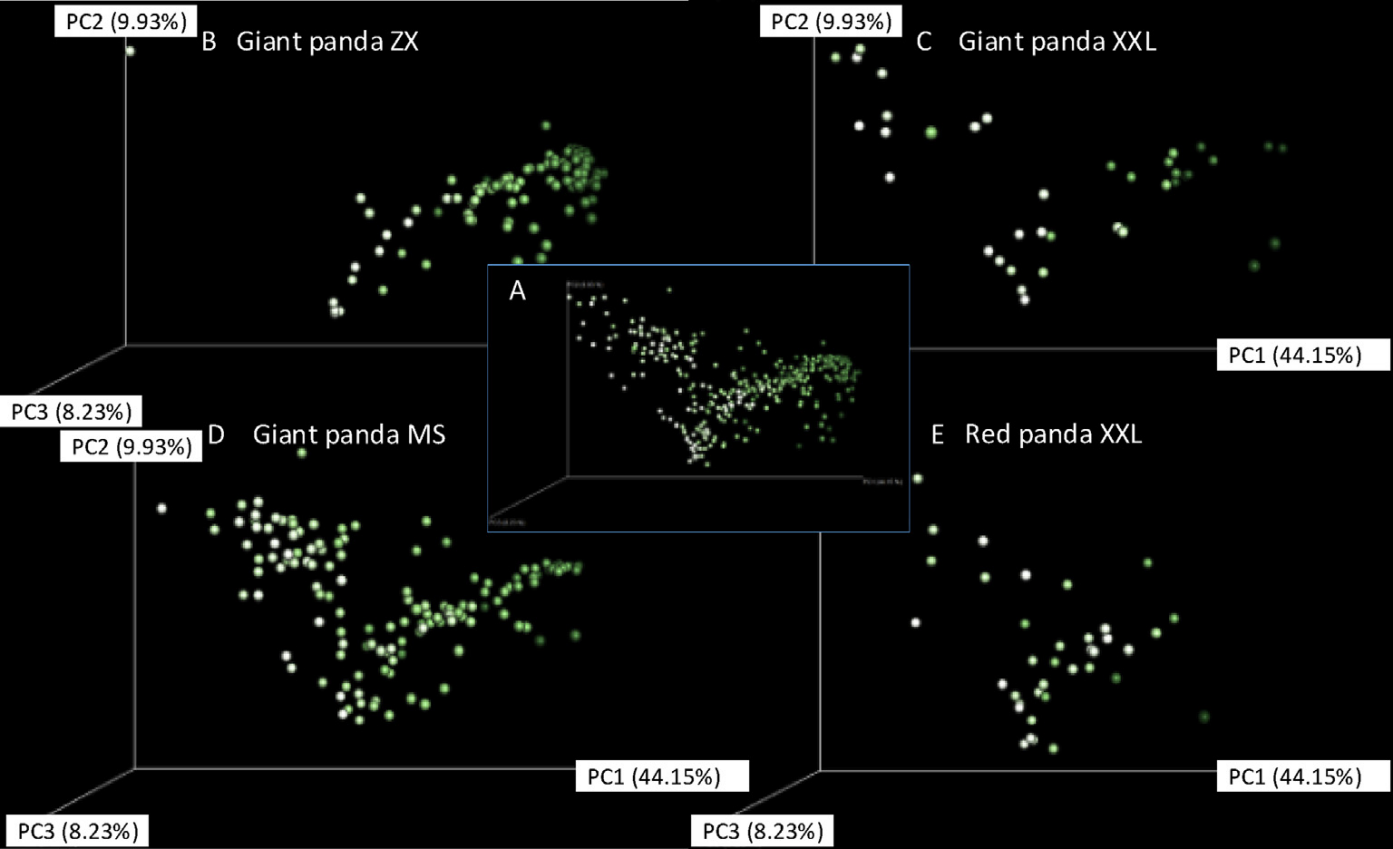
PCoA analysis using Weighted Unifrac distances for panda gut microbiomes: (A) Total pandas, (B) giant panda Zhangxiang ZX, (C) the Xiaoxiangling XXL population, (D), the Minshan MS population and (E), and the Xiaoxiangling red panda population. Each dot represents one fecal sample. Light grey to dark green represents the abundance of Firmicutes from 0.00015 to 0.99396.

**Fig. 4:**
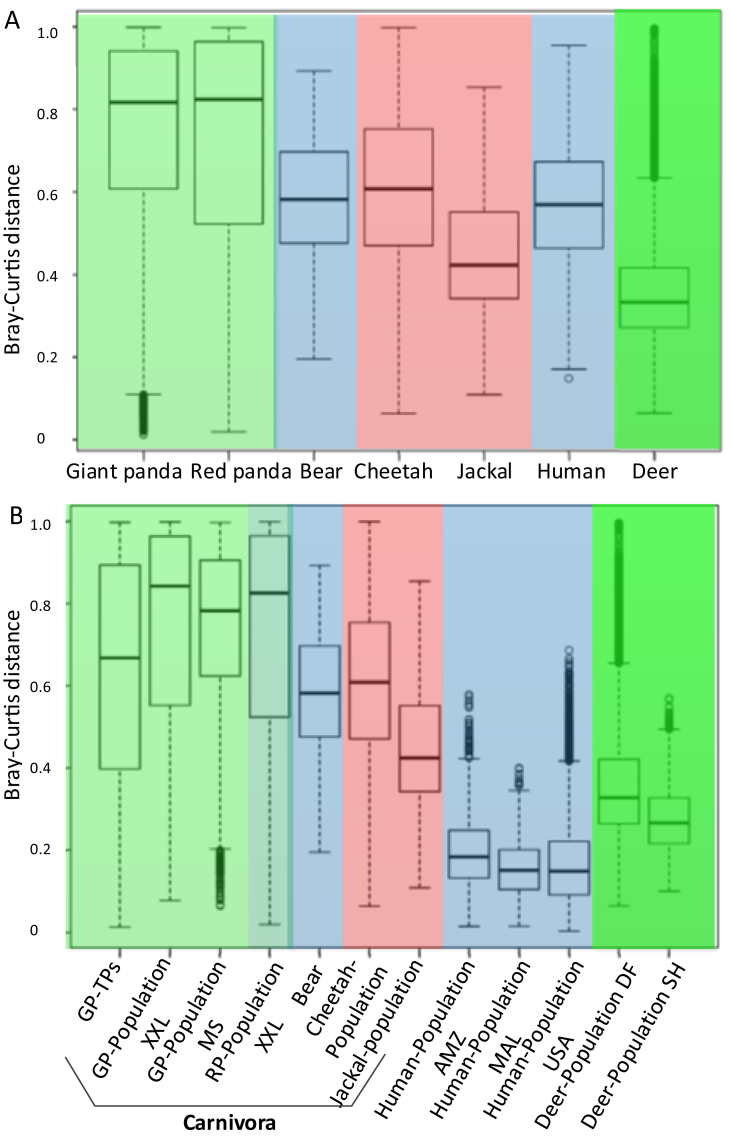
Bray-Curtis dissimilarity using gut microbiome species among the giant panda, red panda, deer, human (Yatsunenko et al., 2012), cheetah (Menke et al., 2014), black-backed jackal (Menke et al., 2014), and black bear (Song et al., 2017). GP, giant panda; RP, red panda; AMZ, human Amazons; MAL, human Malawi. DF, Dafeng Milu Natural Reserve; SH, Shishou Milu Natural Reserve. Bear, black bear from three conservation centers in China.

Regression analysis using core gut microbiome Shannon diversity indicated a more discrete distribution in pandas (Fig. 2A and B) compared with deer or humans (Yatsunenko et al., 2012) (Fig. 2C–F), suggesting high variation in the panda core gut microbiome. In giant pandas, most median types (in the range of high Firmicutes and high Proteobacteria) maintain a high Shannon diversity. Weighted unifrac PCoA analysis indicates dissimilarity between high Firmicutes and high Proteobacteria fecal samples (Fig. 3). Both the weighted unifrac and unweighted unifrac PCoA displayed the admixture pattern among giant panda and red panda samples, indicating the high similarity in their gut microbial community (Fig. 3A and Fig. S2). Significantly high Bray-Curtis dissimilarity using gut microbial genera (genus level in QIIME taxonomy level 6) was uncovered when giant panda and red panda samples were compared with those from deer, humans, cheetah (Menke et al., 2014), black-backed jackal (Menke et al., 2014), and black bear (Song et al., 2017) (Fig. 4A, Welch Two Sample t-test *p* < 0.001). Then, at the population level, the dissimilarity within giant panda or red panda populations were higher than those in the other mammal populations (Fig. 4B). These findings further indicated high variation in panda gut microbiomes.

### 2.4 The functional dissimilarity between high Firmicutes (GP-HF) and high Proteobacteria (GP-HP) gut microbiomes of giant pandas

**Fig. 5:**
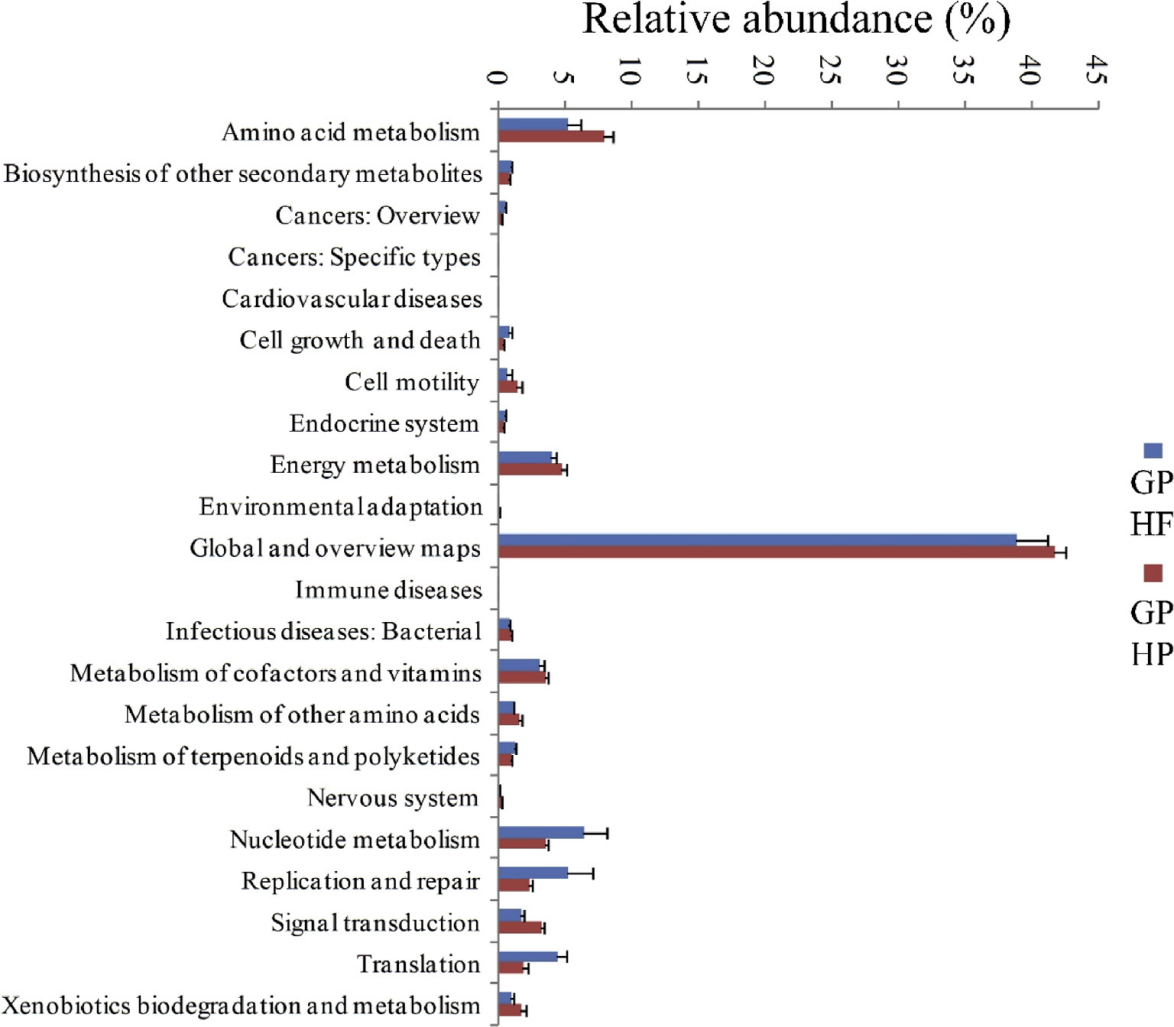
Metagenomic analysis of gut microbial communities from GP-HP and GP-HF. Significantly different (Welch's *t* test, p < 0.05) relative abundances of ‘KEGG level 2’ pathways between HF (high proportion Firmicutes type) and HP (low proportion Firmicutes type).

**Fig. 6:**
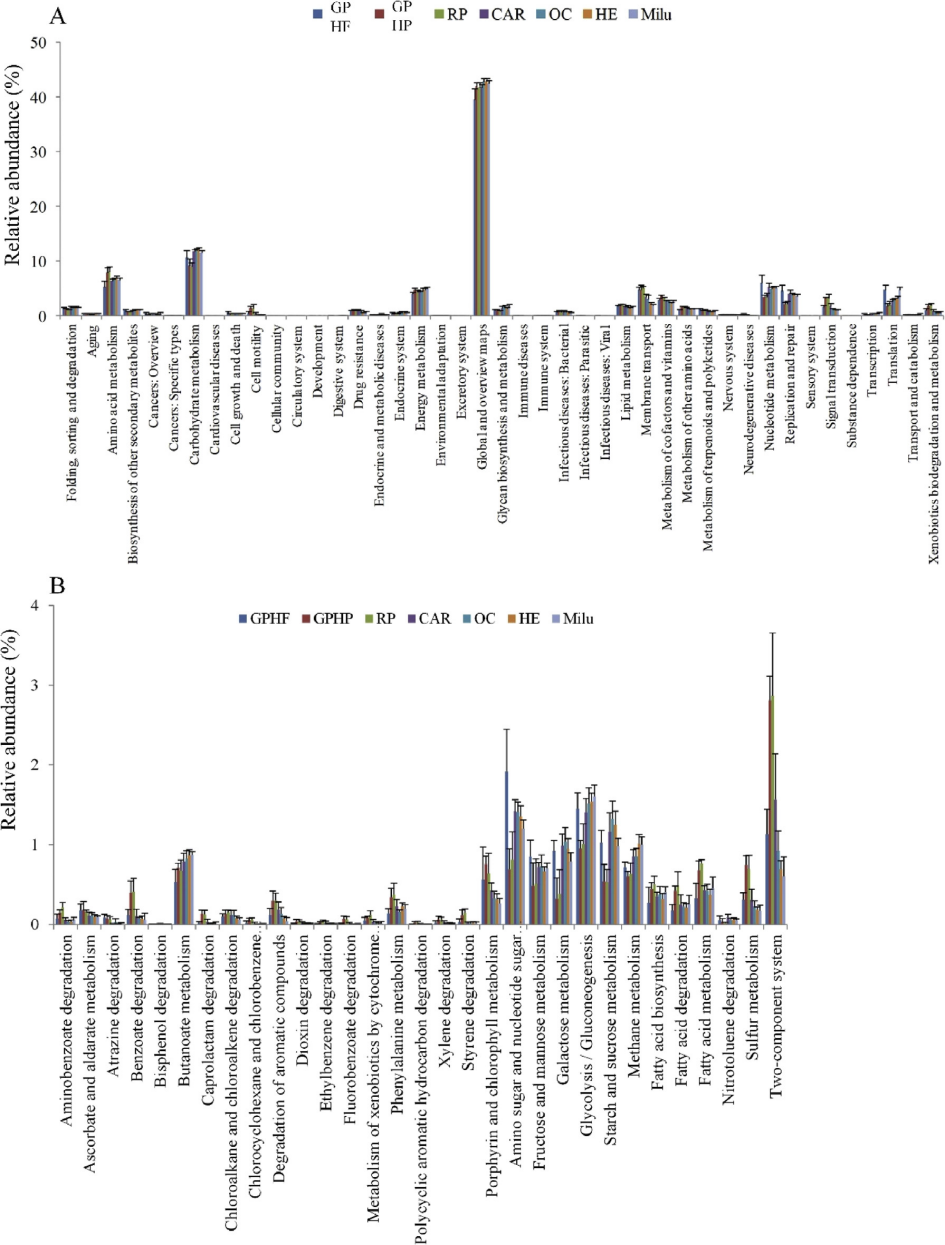
Metagenomic analysis of gut microbial communities from giant pandas, red pandas, and other mammals. (A) Significantly different (ANOVA, p < 0.05) relative abundances of KEGG level 2 pathways among 94 gut bacterial metagenomes (including 19 from giant pandas, 6 from red pandas, 30 from Milu (Père David's deer), and 39 other mammalian gut microbiome metagenomes (Muegge et al., 2011)). (B) Enrichment of metabolic pathways related to xenobiotic biodegradation and metabolism, amino acid metabolism, metabolism of cofactors and vitamins and lipid metabolism in GP-HP and red pandas. RP, red pandas; CAR, carnivorous mammals; OC, omnivorous mammals; HE, herbivorous mammals. Milu, Père David's deer.

We compared functional dissimilarity using KEGG analysis of 25 gut microbial community metagenomes including 19 for giant pandas (three of which were from a previously published dataset (Zhu et al., 2011)) representing seven high Firmicutes and ten high Proteobacteria community types, and six metagenomes from red pandas, five of which were high Proteobacteria community types (Table S3). Analyses comparing the relative proportions of genes encoding for enzymes involved in the functional pathways at ‘KEGG level 2’ revealed enrichment of functions in community types and significant differences between GP-HP and GP-HF community types. Xenobiotics biodegradation and metabolism, Amino acid metabolism, metabolism of cofactors and vitamins, metabolism of other amino acids were particularly enriched in GP-HP microbial communities (Fig. 5). Further, the relative proportions of genes encoding for enzymes involved in 150 functional pathways were significantly different among groups at ‘KEGG level 3’. Of these, 89 were significantly higher in the GP-HP type communities than those of the GP-HF, and included functions in pathways involved in xenobiotic metabolism and degradation of compounds such as aminobenzoate, benzoate, chlorocyclohexane, chlorobenzene, aromatic compounds, dioxin, ethylbenzene, fluorobenzoate, geraniol, limonene and pinene, and polycyclic aromatic hydrocarbons. Sixty-one enzymes were significantly enriched in HF gut microbial communities and included pathways involved in the metabolism of carbohydrates such as starch, sucrose, amino sugars, nucleotide sugars, fructose, mannose, and galactose, in addition to enzymes generally involved in glycolysis and/or gluconeogenesis (Fig. S3). After comparing giant panda and read panda metagenomes to 30 Milu (*Elaphurus davidianus*) gut community metagenomes produced here, in addition to 39 other mammalian gut community metagenomes (Muegge et al., 2011) (including seven carnivores, 11 omnivores and 21 herbivores), we also found that gut community functions of GP-HP and red panda were enriched in xenobiotic biodegradation and metabolism, amino acid metabolism, metabolism of cofactors and vitamins, and lipid metabolisms and GP-HF were enriched in carbohydrate metabolism. The proportions of genes involved in carbohydrate metabolism were highest in herbivorous mammal gut microbial communities (Fig. 6A and B), which might be correlated their diet, having high proportion of cellulose and hemicellulos.

## 3 Discussion

### 3.1 Reconsidering the co-evolution between host diet and their gut microbes

Panda gut microbiome, especially giant panda, showed high variation and instability compared with the gut microbiomes of deer, humans, black bear, cheetah and black-backed jackal. This is in contrast to the primary pattern found in many animals of stable core gut microbiomes under similar diets and similar diets leading to similar gut microbial communities within the same host species level (Lozupone et al., 2012; Faith et al., 2013; David et al., 2014; Gerber, 2014; Sullam et al., 2015; Weingarden et al., 2015; Kwong and Moran, 2016; McNally and Brown, 2016; Tinker and Ottesen, 2016; Menke et al., 2017). Diet and phylogeny are the main factors impacting gut microbiomes (Ley et al., 2006, 2008). However, giant panda gut microbial system showed that same long-term diet does not always lead to similar or stable gut microbiomes within the same host species; perturbation factors or selective pressure may lead to microbial instability (Kostic et al., 2013).

This high instability and dissimilarity in panda gut microbial communities might reflect, in part, a potentially unstable gut environment (perturbation) due to their typical carnivorous gastrointestinal system, but herbivorous diet. Short digestive tracts, brief digestion times and fast intestinal peristalsis may result in higher oxygen concentrations that select for the growth of aerobes and facultative anaerobes (Zhang et al., 1995), such as the Pseudomonadaceae. Compared to individuals studied here and elsewhere including captive giant pandas (Zhang et al., 1995; Ley et al., 2008; Zhu et al., 2011; Xue et al., 2015), captive red pandas (Ley et al., 2008) and other herbivorous mammals (Ley et al., 2008), the mean proportion of Pseudomonadaceae was the highest in our wild red panda samples. Pseudomonadaceae are capable of aerobic metabolism (oxidase positive) and are widely distributed among environments (Cornelis, 2008).

### 3.2 The functional divergence of giant panda gut microbiome in high variation of gut microbiome under dietary plant toxins pressures

For controlled human, some phylum-level variation (such as Firmicutes/Bacteroides shift) were earlier observed; but the variation of microbial compositions is to great extent redundant and the related functional characteristics was non-redundant or similar in different individuals (Lozupone et al., 2012). Compared to humans and other mammal species, the gut microbiomes of giant panda displayed the relatively high variation and shift. Here, when compared the KEGG functional pathways between GP-HP and GP-HF groups, we found the high dissimilarity on the functional level. For example, dominant Proteobacteria families, such as the Pseudomonadaceae, were positively and significantly correlated with most pathways involved in xenobiotic biodegradation and metabolism, amino acid metabolism and metabolism of cofactors and vitamins, but were significantly and negatively correlated with most carbohydrate metabolism pathways. Dominant Firmicutes families, including the Clostridiaceae, Lactobacillaceae and Ruminococcaceae exhibited the converse patterns (Fig. S4). Bamboo is especially rich in plant secondary metabolites (e.g. allied phenolic, polyphenolic compounds, and terpenoids) (Keski-Saari et al., 2008; Satya et al., 2010; Choudhury et al., 2012)) and carbohydrates (e.g. cellulose and hemicellulose). Thus, GP-HP might play important role in secondary metabolites (including Xenobiotics), and however, GP-HF might focus on cellulose digestion, which supported by our previous findings that gut microbiome with high proportion of Clostridiaceae from Firmicutes are involved in cellulose digestion (Zhu et al., 2011).

Bamboo is especially rich in plant secondary metabolites (e.g. allied phenolic, polyphenolic compounds, terpenoids, aromatic compounds, cyanide compounds and cellulose and hemicellulose) (Schaller, 1985; Keski-Saari et al., 2008). Aerobic and facultative anaerobic species may be capable of degradation of such secondary metabolites. In some insect larval symbionts, *Pseudomonas* is responsible for plant defense suppression (Chung et al., 2013). Thus, there are trade-offs between Pseudomonadaceae and Clostridiaceae in the panda gut microbiome: Pseudomonadaceae plays an important role in secondary metabolites (including xenobiotics) and obligate anaerobic Clostridiaceae may focus on cellulose digestion. This is supported by the previous work on showing (1) increasing the utilization of fermentable sugars during the enzymatic hydrolysis of microcrystalline cellulose in corncobs (Luo et al., 2013), (2) a high proportion of Clostridiaceae from Firmicutes involved in cellulose digestion in the panda gut microbiome (Zhu et al., 2011), and (3) high proportion of Pseudomonadaceae in the panda gut microbiome associated with potential degradation of cyanide compounds (Zhu et al., 2018). Median type fecal samples (40% of total panda fecal samples) had a relatively high Shannon diversity and might reflect some equilibrium across secondary compounds degradation (detoxification) and carbohydrate metabolism (e.g. cellulose digestion). Some Pseudomonas species are able to consume aromatic compounds in the presence of glucose (Perchet et al., 2008), which is one of the metabolites of cellulose digestion. Thus, this might be one explanation for the co-existence of Pseudomonadaceae and Clostridiaceae in the panda gut.

### 3.3 The other factors might contribute to the variations of gut microbial compositions

Seasonal variation in nutrient utilization may shape gut microbiome structure and function in wild giant pandas (Wu et al., 2017). It has been wildly accepted that the major component in the panda feed are bamboo parts (e.g., bamboo stems, leaves, and shoots). Here, we have collected fecal samples from different season in XXL wild giant panda population, which also might contribute to the variations of gut microbial compositions. Thus, the variation in feed composition (regional/seasonal) would cause the perturbation in the panda gut microbiome. In future, the investigation of the long-term relationship between panda gut microbiome and bamboo nutrition in the wild habitats will be one of interesting studies in panda microbial ecology.

## 4 Conclusion

Therefore, we speculated that the variation in oxygen concentrations and potential plant secondary metabolites or xenobiotic would likely lead to the big change in panda gut microbial communities. In the future, if possible, combining the nutrition and host physiological data under the long-term monitoring (individual and population level) will provide a more in-depth understanding of the coevolution between giant panda and their gut microbes.

## 5 Materials and methods

### 5.1 Sample collection, DNA extraction and 16S rRNA gene sequencing

Fresh feces from giant pandas and red pandas in the Xiaoxiangling (XXL) Mountains were collected from 2012–2016, including samples from wild populations and five translocated individuals (Luxin, Zhangxiang, Taotao, Huajiao and Xuexue). Main dietary bamboos include *Bashania spanostachya Yi* and *Yushania lineolate*. Feces were collected directly from GPS-collared individuals by a monitoring team. Fresh feces were frozen and then shipped on dry ice to the laboratory for analysis. Fresh fecal samples of giant pandas in the Minshan (MS) populations were collected in June 2012. Due to the logistics of collecting samples from MS populations, fresh samples were preserved in alcohol. Main dietary bamboos include *Fargesia denudate*, *F. nitida*, *F. scabtida*, and *F. rufa*. All samples were frozen and shipped on dry ice to the laboratory for analysis (Table S1). Fresh fecal samples from Père David's deer were collected during long-term field monitoring from 2011–2014. Fresh fecal samples (n = 245) were collected from the Dafeng Milu National Preserve (Wang et al., 2019), and 70 samples were collected from the Shishou Milu National Preserve in November 2014. All samples were frozen and shipped as described above (Table S2).

Total DNA was extracted from fecal samples using QIAGEN DNA stool kits (QIAGEN, Germany) according to the manufacturer's protocols. The V4–V5 region of the bacterial 16S ribosomal RNA gene was amplified by PCR (95 °C for 2 min, followed by 25 cycles at 95 °C for 30 s, 55 °C for 30 s, and 72 °C for 30 s and a final extension step of 72 °C for 5 min) using the 515F (5′-barcode-GTGCCAGCMGCCGCGG-3′) and 907R (5′-CCGTCAATTCMTTTRAGTTT-3′) primers, where the barcode is an eight bp sequence unique to each sample. PCR reactions were performed in triplicate in 20 μL final volumes using mixtures containing 4 μL of 5 × FastPfu Buffer, 2 μL of 2.5 mM dNTPs, 0.8 μL of each primer (5 μM), 0.4 μL of FastPfu Polymerase, and 10 ng of template DNA. Amplicons were extracted from 2% agarose gels and purified using the AxyPrep DNA Gel Extraction Kit (Axygen Biosciences, Union City, CA, USA) according to the manufacturer's instructions and quantified using QuantiFluor™ -ST (Promega, USA).

#### 5.1.1 Library construction and sequencing

Purified PCR products were quantified using a Qubit®3.0 analyzer (Life Invitrogen) and 24 amplicons with different barcodes were mixed in equal proportions for a single pooled preparation. Pooled DNA products were used to construct an Illumina paired-end library following Illumina's genomic DNA library preparation procedure. The amplicon library was paired-end sequenced (2 × 250 bp) on an Illumina MiSeq platform (Shanghai BIOZERON) according to standard protocols.

#### 5.1.2 Processing of sequencing data

Pairwise ends raw fastq files were demultiplexed and quality-filtered using QIIME v1.9 (Caporaso et al., 2010) and the following criteria: (i) the 250 bp reads were truncated at any site that had an average quality score <20 over a 10 bp sliding window and truncated reads that were shorter than 50 bp were discarded; (ii) exact barcode matching, two nucleotide mismatches allowed in primer sequences, and the removal of reads containing ambiguous characters; and (iii) only sequences that contained overlap greater than 10 bp were assembled. Reads which could not be assembled were discarded. Sequences were clustered into 97% OTUs through uclust_ref and assigned to a taxonomic level (phylum, class, order, family, and genus) against Silver database using QIIME v1.9 (Caporaso et al., 2010). Single_rarefaction.py in QIIME was used to perform rarefaction on the OTU table (6456 sequences to subsample per sample) for downstream analysis. The relative abundance in each fecal samples were calculated based on this rarefaction OTU table. For methodology, referring to Raup (1975), taxonomic diversity estimation used rarefaction (Raup, 1975). To assess differences in community composition, PCoA (weighted unifrac and unweighted unifrac distance) was also conducted in QIIME v1.9 (Caporaso et al., 2010). Vegan in R program was used to calculate Bray-Curtis distances using microbial genera among each group (Team, 2000; Oksanen et al., 2007). Non-parameter Welch Two Sample t-test used to test the significant difference in the Bray-Curtis distances between giant panda or red panda with other mammal.

In addition, to evaluate the dynamics on the gut microbiomes of giant pandas and red pandas, we chose the gut microbiomes from several mammal species as the control groups: (1) we wanted to use some published gut microbiome data from different kind of mammals representing the different diet, such as humans (Yatsunenko et al., 2012) (omnivore, Primates), deer (typical herbivore), cheetah and jackal (Menke et al., 2014) (carnivore, same phylum with giant pandas and red pandas), and black bear (Song et al., 2017) (omnivorous diet, phylogenetically close to giant panda, and both of them belong to Ursidae); (2) These studies use the similar 16S V4 region; (3) These studies have the detailed in sample information; and (4) Pere David deer (our dataset, typical herbivore, no transition on the diet changes liking in pandas) has the detailed sample information for time.

### 5.2 Metagenomic sequencing of the panda microbial communities

Metagenomic sequencing (including community DNA from 16 giant panda samples, six red panda samples (Table S3), and 30 deer samples (Table S4)) was performed by BIOZERON (Shanghai, China). A library was constructed with an average insert size of 450 bp for each sample. Sequencing was performed using an Illumina Hiseq 2500 platform. Illumina GA Hiseq reads were filtered using custom Perl scripts and publicly available software to remove (i) all reads less than 50 bp in length, (ii) reads with degenerate bases (N's), and (iii) all duplicates defined as sequences whose initial 20 nucleotides were identical and shared an overall identity of >97% throughout the length of the shortest read. Raw short reads were compared against the host genome to facilitate the removal of host genomic sequences. The resultant clean, high-quality reads were assembled to generate contigs using the SOAPdenovo assembler (Li et al., 2010). Taxonomic classification of predicted gene sequences was determined with MEGAN5 (Huson et al., 2007). CD-HIT was used to construct non-redundant gene sets with less than 90% overlap and less than 95% shared sequence identity (Li and Godzik, 2006). The SOAPdenovo assembler was used to generate a gene profile for each metagenomics sample (Li et al., 2010). Based on these gene profiles, non-redundant gene sequences were searched against the Kyoto Encyclopedia of Genes and Genomes (KEGG) database using BLASTP (Altschul et al., 1997). A sequence read was annotated as the most optimal hit in the database if (i) the E-value was <10^−5^, (ii) the bit score was >50, and (iii) the alignment was at least 50% identical between the query and subject. In the event that two entries in the database had equivalent BLAST scores and were both deemed best hits, the read was annotated with both entries. The KEGG orthology, enzyme commission, and KEGG pathways associated with each sequence were determined, and converted to a QIIME-readable biom format. The taxonomic distribution of metagenomic reads were again determined using MEGAN (Huson et al., 2007). Non-redundant gene sequences were searched against the NCBI non-redundant protein database using BLASTX. The significantly differential abundant feature detection of KEGG pathways from all data were used in further Lefse (linear discriminant analysis effect size) analysis (Segata et al., 2011). We also incorporated previously published metagenomic datasets (Muegge et al., 2011; Zhu et al., 2011), and the relative abundance of KEGG pathways from all data were used in further STAMP analysis (Parks et al., 2014). The Spearman correlation of bacteria abundance with KEGG pathways was calculated by Correlation. R (http://userweb.eng.gla.ac.uk/umer.ijaz).

## Declarations

### Author contribution statement

Ran Yao, Zhisong Yang, Zheng Zhang, Ting Hu: Performed the experiments; Wrote the paper.

Hua Chen: Analyzed and interpreted the data; Contributed reagents, materials, analysis tools or data; Wrote the paper.

Feng Huang, Xiaodong Gu, Xuyu Yang: Contributed reagents, materials, analysis tools or data; Wrote the paper.

Guoqing Lu: Analyzed and interpreted the data; Wrote the paper.

Lifeng Zhu: Conceived and designed the experiments; Performed the experiments; Analyzed and interpreted the data; Contributed reagents, materials, analysis tools or data; Wrote the paper.

### Funding statement

This work was supported by grants from the National Natural Science Foundation of China - Outstanding Young Foundation (31222009), National Natural Science Foundation of China (31570489, 31741112), National Key Programme of Research and Development, Ministry of Science and Technology (2016YFC0503200) of China, the Priority Academic Program Development of Jiangsu Higher Education Institutions (PAPD) of China, and the Reintroduction Program of Giant Pandas of China.

### Competing interest statement

The authors declare no conflict of interest.

### Additional information

Data associated with this study has been deposited at NCBI under the accession number PRJNA497650.

Supplementary content related to this article has been published online at https://doi.org/10.1016/j.heliyon.2019.e02480.

## Appendix A Supplementary data

Zhu_SI Zhu_SI
