# Supplementary material for: Are the gut microbial systems of giant pandas unstable?
Source: Heliyon. 2019 Sep 17;5(9):e02480. doi: 10.1016/j.heliyon.2019.e02480 (PMC6819816; doi:10.1016/j.heliyon.2019.e02480)
Supplement: Zhu_SI [file mmc1.docx]

Supplementary Materials

**Table S1** Detailed information (collection time and groups) of panda fecal samples

| Sample ID | Group1 | Group2 | Collection time | Bacteria group | Clean-read |
| --- | --- | --- | --- | --- | --- |
| 15 | TP | HJ | 12/12/2015 | HJXX-HP | 36997 |
| 16 | TP | HJ | 11/29/2015 | HJXX-M | 33098 |
| 34 | TP | HJ | 01/21/2016 | HJXX-M | 29610 |
| 35 | TP | HJ | 03/26/2016 | HJXX-HF | 23115 |
| 43 | TP | HJ | 12/01/2015 | HJXX-M | 29839 |
| 54 | TP | HJ | 12/09/2015 | HJXX-M | 37094 |
| 59 | TP | HJ | 11/25/2015 | HJXX-M | 14970 |
| 10zl | TP | HJ | 03/26/2016 | HJXX-HF | 33428 |
| 9zl | TP | HJ | 11/22/2015 | HJXX-HP | 20764 |
| 1 | Wild-TP | LX | 09/14/2013 | LXTT-HP | 13831 |
| 2 | Wild-TP | LX | Unclear/2013 | LXTT-HF | 12203 |
| 9 | Wild-TP | LX | 09/14/2013 | LXTT-HP | 29959 |
| 13 | Wild-TP | LX | 08/03/2014 | LXTT-HP | 32024 |
| 48 | Wild-TP | LX | 08/03/2014 | LXTT-HP | 34258 |
| 12zl | Wild-XXL | XXL | 08/03/2014 | XXL-HP | 26102 |
| CDXM15 | Wild-TP | LX | 04/16/2014 | LXTT-M | 15423 |
| CDXM37 | Wild-TP | LX | 05/26/2014 | LXTT-HP | 15559 |
| CDXM42 | Wild-TP | LX | 04/10/2014 | LXTT-HP | 19428 |
| SHI02 | Wild-TP | LX | unclear/2013 | LXTT-M | 13617 |
| SHI08 | Wild-TP | LX | 01/19/2013 | LXTT-HF | 14228 |
| SHI14 | Wild-TP | LX | 05/26/2013 | LXTT-M | 16418 |
| GS1 | Wild-MS | MS | 06-07/2012 | MS-HP | 24437 |
| GS10 | Wild-MS | MS | 06-07/2012 | MS-HP | 26093 |
| GS21 | Wild-MS | MS | 06-07/2012 | MS-M | 36884 |
| GS22 | Wild-MS | MS | 06-07/2012 | MS-HP | 27872 |
| GS25 | Wild-MS | MS | 06-07/2012 | MS-HP | 32800 |
| GS28 | Wild-MS | MS | 06-07/2012 | MS-HF | 31909 |
| GS30 | Wild-MS | MS | 06-07/2012 | MS-HP | 23308 |
| GS35 | Wild-MS | MS | 06-07/2012 | MS-HP | 23189 |
| GS45 | Wild-MS | MS | 06-07/2012 | MS-HP | 39803 |
| GS48 | Wild-MS | MS | 06-07/2012 | MS-HP | 23933 |
| GS56 | Wild-MS | MS | 06-07/2012 | MS-HP | 32082 |
| GS59 | Wild-MS | MS | 06-07/2012 | MS-M | 38942 |
| GS65 | Wild-MS | MS | 06-07/2012 | MS-HP | 37106 |
| GS9 | Wild-MS | MS | 06-07/2012 | MS-HP | 34839 |
| ms02 | Wild-MS | MS | 06-07/2012 | MS-HP | 25652 |
| ms101 | Wild-MS | MS | 06-07/2012 | MS-HP | 30190 |
| MS103 | Wild-MS | MS | 06-07/2012 | MS-HP | 33267 |
| MS106 | Wild-MS | MS | 06-07/2012 | MS-HF | 39879 |
| MS107 | Wild-MS | MS | 06-07/2012 | MS-HF | 26694 |
| ms108 | Wild-MS | MS | 06-07/2012 | MS-HP | 29486 |
| MS111 | Wild-MS | MS | 06-07/2012 | MS-HP | 25647 |
| ms112 | Wild-MS | MS | 06-07/2012 | MS-M | 29874 |
| MS12 | Wild-MS | MS | 06-07/2012 | MS-HP | 35230 |
| MS122 | Wild-MS | MS | 06-07/2012 | MS-HP | 28243 |
| ms128 | Wild-MS | MS | 06-07/2012 | MS-M | 37841 |
| ms135 | Wild-MS | MS | 06-07/2012 | MS-M | 37788 |
| MS139 | Wild-MS | MS | 06-07/2012 | MS-M | 31718 |
| ms144 | Wild-MS | MS | 06-07/2012 | MS-HP | 26690 |
| MS145 | Wild-MS | MS | 06-07/2012 | MS-M | 32312 |
| ms146 | Wild-MS | MS | 06-07/2012 | MS-HP | 39131 |
| MS148 | Wild-MS | MS | 06-07/2012 | MS-HP | 29826 |
| ms152 | Wild-MS | MS | 06-07/2012 | MS-M | 34740 |
| MS156 | Wild-MS | MS | 06-07/2012 | MS-HP | 28586 |
| MS160 | Wild-MS | MS | 06-07/2012 | MS-M | 32496 |
| MS161 | Wild-MS | MS | 06-07/2012 | MS-HP | 35023 |
| ms162 | Wild-MS | MS | 06-07/2012 | MS-M | 28216 |
| MS167 | Wild-MS | MS | 06-07/2012 | MS-M | 35294 |
| MS168 | Wild-MS | MS | 06-07/2012 | MS-HP | 31936 |
| MS173 | Wild-MS | MS | 06-07/2012 | MS-M | 21077 |
| MS174 | Wild-MS | MS | 06-07/2012 | MS-M | 30305 |
| ms187 | Wild-MS | MS | 06-07/2012 | MS-M | 36744 |
| MS201 | Wild-MS | MS | 06-07/2012 | MS-M | 37471 |
| MS217 | Wild-MS | MS | 06-07/2012 | MS-HP | 21190 |
| MS219-kuohao-3.19 | Wild-MS | MS | 06-07/2012 | MS-M | 35817 |
| MS22 | Wild-MS | MS | 06-07/2012 | MS-HP | 31790 |
| MS220 | Wild-MS | MS | 06-07/2012 | MS-M | 33487 |
| MS222 | Wild-MS | MS | 06-07/2012 | MS-HP | 36590 |
| ms223 | Wild-MS | MS | 06-07/2012 | MS-HP | 23609 |
| MS224 | Wild-MS | MS | 06-07/2012 | MS-HF | 37848 |
| MS226 | Wild-MS | MS | 06-07/2012 | MS-HP | 25067 |
| MS227 | Wild-MS | MS | 06-07/2012 | MS-HF | 30999 |
| MS228 | Wild-MS | MS | 06-07/2012 | MS-HP | 25408 |
| MS229 | Wild-MS | MS | 06-07/2012 | MS-HF | 29231 |
| MS230 | Wild-MS | MS | 06-07/2012 | MS-M | 20404 |
| MS232 | Wild-MS | MS | 06-07/2012 | MS-M | 28000 |
| ms234 | Wild-MS | MS | 06-07/2012 | MS-M | 21476 |
| MS235 | Wild-MS | MS | 06-07/2012 | MS-M | 23693 |
| MS236 | Wild-MS | MS | 06-07/2012 | MS-M | 28658 |
| ms237 | Wild-MS | MS | 06-07/2012 | MS-HP | 24754 |
| ms239 | Wild-MS | MS | 06-07/2012 | MS-HP | 27773 |
| ms240 | Wild-MS | MS | 06-07/2012 | MS-HF | 32270 |
| MS241 | Wild-MS | MS | 06-07/2012 | MS-HF | 37219 |
| MS242 | Wild-MS | MS | 06-07/2012 | MS-M | 28715 |
| MS243 | Wild-MS | MS | 06-07/2012 | MS-M | 34166 |
| ms244 | Wild-MS | MS | 06-07/2012 | MS-HF | 39717 |
| MS245 | Wild-MS | MS | 06-07/2012 | MS-M | 32364 |
| MS247 | Wild-MS | MS | 06-07/2012 | MS-HF | 37974 |
| ms249 | Wild-MS | MS | 06-07/2012 | MS-HP | 39621 |
| MS254 | Wild-MS | MS | 06-07/2012 | MS-M | 39317 |
| MS255 | Wild-MS | MS | 06-07/2012 | MS-HP | 34476 |
| MS257 | Wild-MS | MS | 06-07/2012 | MS-HP | 26890 |
| MS260 | Wild-MS | MS | 06-07/2012 | MS-M | 26889 |
| MS266 | Wild-MS | MS | 06-07/2012 | MS-HP | 34153 |
| MS270 | Wild-MS | MS | 06-07/2012 | MS-HP | 23434 |
| ms271 | Wild-MS | MS | 06-07/2012 | MS-M | 31116 |
| MS272 | Wild-MS | MS | 06-07/2012 | MS-M | 26574 |
| ms273 | Wild-MS | MS | 06-07/2012 | MS-HP | 38739 |
| ms275 | Wild-MS | MS | 06-07/2012 | MS-M | 34056 |
| MS276 | Wild-MS | MS | 06-07/2012 | MS-M | 33357 |
| MS277 | Wild-MS | MS | 06-07/2012 | MS-HP | 34038 |
| ms278 | Wild-MS | MS | 06-07/2012 | MS-M | 26969 |
| ms28 | Wild-MS | MS | 06-07/2012 | MS-M | 39899 |
| ms280 | Wild-MS | MS | 06-07/2012 | MS-HP | 36056 |
| MS281 | Wild-MS | MS | 06-07/2012 | MS-HP | 30393 |
| MS282 | Wild-MS | MS | 06-07/2012 | MS-HP | 20025 |
| MS284 | Wild-MS | MS | 06-07/2012 | MS-HP | 33846 |
| MS285 | Wild-MS | MS | 06-07/2012 | MS-HP | 32261 |
| MS286 | Wild-MS | MS | 06-07/2012 | MS-HF | 34737 |
| MS289 | Wild-MS | MS | 06-07/2012 | MS-M | 20163 |
| MS290-kuohao-3.20 | Wild-MS | MS | 06-07/2012 | MS-M | 33588 |
| ms291 | Wild-MS | MS | 06-07/2012 | MS-HP | 28145 |
| ms292 | Wild-MS | MS | 06-07/2012 | MS-M | 33304 |
| ms295 | Wild-MS | MS | 06-07/2012 | MS-M | 29278 |
| MS296 | Wild-MS | MS | 06-07/2012 | MS-HF | 20366 |
| ms297 | Wild-MS | MS | 06-07/2012 | MS-M | 25890 |
| MS298 | Wild-MS | MS | 06-07/2012 | MS-HP | 24484 |
| MS30 | Wild-MS | MS | 06-07/2012 | MS-HF | 35499 |
| MS302 | Wild-MS | MS | 06-07/2012 | MS-HP | 35766 |
| MS303 | Wild-MS | MS | 06-07/2012 | MS-HF | 24921 |
| ms304 | Wild-MS | MS | 06-07/2012 | MS-HP | 24160 |
| ms305 | Wild-MS | MS | 06-07/2012 | MS-HP | 31283 |
| MS306 | Wild-MS | MS | 06-07/2012 | MS-HF | 23055 |
| ms307 | Wild-MS | MS | 06-07/2012 | MS-M | 31607 |
| MS309 | Wild-MS | MS | 06-07/2012 | MS-HP | 38708 |
| MS30-kuohao-3.19 | Wild-MS | MS | 06-07/2012 | MS-M | 36113 |
| MS310 | Wild-MS | MS | 06-07/2012 | MS-M | 36580 |
| ms311 | Wild-MS | MS | 06-07/2012 | MS-M | 24298 |
| MS34 | Wild-MS | MS | 06-07/2012 | MS-HP | 37393 |
| MS38 | Wild-MS | MS | 06-07/2012 | MS-HP | 37247 |
| MS44 | Wild-MS | MS | 06-07/2012 | MS-HP | 37635 |
| MS57 | Wild-MS | MS | 06-07/2012 | MS-M | 32253 |
| MS58 | Wild-MS | MS | 06-07/2012 | MS-M | 36926 |
| MS63 | Wild-MS | MS | 06-07/2012 | MS-HP | 35663 |
| MS64 | Wild-MS | MS | 06-07/2012 | MS-HP | 39837 |
| MS70 | Wild-MS | MS | 06-07/2012 | MS-M | 33930 |
| ms70 | Wild-MS | MS | 06-07/2012 | MS-M | 35238 |
| MS75 | Wild-MS | MS | 06-07/2012 | MS-M | 37986 |
| MS76 | Wild-MS | MS | 06-07/2012 | MS-M | 26766 |
| MS80 | Wild-MS | MS | 06-07/2012 | MS-M | 39648 |
| ms80 | Wild-MS | MS | 06-07/2012 | MS-HP | 39925 |
| MS86 | Wild-MS | MS | 06-07/2012 | MS-HF | 20687 |
| ms88 | Wild-MS | MS | 06-07/2012 | MS-M | 23011 |
| MS92 | Wild-MS | MS | 06-07/2012 | MS-M | 39303 |
| MS94 | Wild-MS | MS | 06-07/2012 | MS-M | 32397 |
| MS95 | Wild-MS | MS | 06-07/2012 | MS-HP | 26175 |
| MS99 | Wild-MS | MS | 06-07/2012 | MS-HP | 32870 |
| SCMS100 | Wild-MS | MS | 06-07/2012 | MS-HF | 39263 |
| SCMS102 | Wild-MS | MS | 06-07/2012 | MS-M | 27241 |
| SCMS108 | Wild-MS | MS | 06-07/2012 | MS-M | 22364 |
| SCMS109 | Wild-MS | MS | 06-07/2012 | MS-HP | 27835 |
| SCMS119 | Wild-MS | MS | 06-07/2012 | MS-HP | 23224 |
| SCMS140 | Wild-MS | MS | 06-07/2012 | MS-HF | 36843 |
| SCMS142 | Wild-MS | MS | 06-07/2012 | MS-M | 33672 |
| SCMS27 | Wild-MS | MS | 06-07/2012 | MS-HF | 21051 |
| SCMS71 | Wild-MS | MS | 06-07/2012 | MS-HP | 27500 |
| SCMS78 | Wild-MS | MS | 06-07/2012 | MS-M | 20361 |
| SCMS82 | Wild-MS | MS | 06-07/2012 | MS-HP | 37315 |
| SCMS90 | Wild-MS | MS | 06-07/2012 | MS-M | 32958 |
| SM150-kuohao-3.22 | Wild-MS | MS | 06-07/2012 | MS-M | 37929 |
| 26 | TP | TT | 11/22/2014 | LXTT-M | 35976 |
| 28 | TP | TT | 03/15/2015 | LXTT-HF | 37966 |
| 38 | TP | TT | 11/22/2014 | LXTT-HF | 23667 |
| 45 | TP | TT | 08/12/2015 | XXL-HF | 38896 |
| 50 | TP | TT | 05/11/2015 | LXTT-HF | 23565 |
| 53 | TP | TT | 04/30/2014 | LXTT-HF | 33372 |
| 56 | TP | TT | 07/20/2015 | LXTT-HP | 38195 |
| CDXM06 | TP | TT | 11/20/2013 | LXTT-HF | 17267 |
| SHI01 | TP | TT | 03/07/2013 | LXTT-HP | 16625 |
| SHI04 | TP | TT | 01/17/2013 | LXTT-HF | 17636 |
| 21 | TP | XX | 11/19/2014 | HJXX-HP | 24973 |
| 5 | Wild-XXL | XXL | 10/25/2013 | XXL-HP | 22662 |
| 6 | Wild-XXL | XXL | 10/25/2013 | XXL-M | 20503 |
| 7 | Wild-XXL | XXL | 10/23/2013 | XXL-HP | 21735 |
| 8 | Wild-XXL | XXL | 10/25/2013 | XXL-HP | 25154 |
| 10 | Wild-XXL | XXL | 09/28/2013 | XXL-HF | 11767 |
| 14 | Wild-XXL | XXL | 10/25/2013 | XXL-HP | 30204 |
| 20 | Wild-XXL | XXL | 08/10/2013 | XXL-HP | 32223 |
| 25 | Wild-XXL | XXL | 10/24/2013 | XXL-HP | 20381 |
| 29 | Wild-XXL | XXL | 02/28/2013 | XXL-HP | 32140 |
| 46 | Wild-XXL | XXL | 10/21/2014 | XXL-HP | 21905 |
| 3zl | Wild-XXL | XXL | 09/28/2013 | XXL-HF | 21667 |
| 4zl | Wild-XXL | XXL | 05/16/2014 | XXL-HF | 37232 |
| 5zl | Wild-XXL | XXL | 05/2014 | XXL-HF | 34043 |
| CDXM17 | Wild-XXL | XXL | 04/08/2014 | XXL-M | 13683 |
| CDXM18 | Wild-XXL | XXL | 04/06/2014 | XXL-HF | 18269 |
| CDXM19 | Wild-XXL | XXL | 04/08/2014 | XXL-M | 11113 |
| CDXM20 | Wild-XXL | XXL | 03/27/2014 | XXL-M | 17322 |
| CDXM21 | Wild-XXL | XXL | 02/08/2014 | XXL-M | 13471 |
| CDXM22 | Wild-XXL | XXL | 04/08/2014 | XXL-HF | 16528 |
| CDXM23 | Wild-XXL | XXL | 03/27/2014 | XXL-HP | 17549 |
| CDXM24 | Wild-XXL | XXL | 03/27/2014 | XXL-HP | 14459 |
| CDXM25 | Wild-XXL | XXL | 03/16/2014 | XXL-HF | 17484 |
| CDXM26 | Wild-XXL | XXL | 04/08/2014 | XXL-HP | 18334 |
| CDXM27 | Wild-XXL | XXL | 03/16/2014 | XXL-HP | 13734 |
| CDXM28 | Wild-XXL | XXL | 03/27/2014 | XXL-HF | 17351 |
| CDXM29 | Wild-XXL | XXL | 04/08/2014 | XXL-HF | 10602 |
| CDXM30 | Wild-XXL | XXL | 04/08/2014 | XXL-M | 13090 |
| CDXM32 | Wild-XXL | XXL | 04/08/2014 | XXL-M | 14837 |
| CDXM33 | Wild-XXL | XXL | 03/27/2014 | XXL-HP | 14054 |
| CDXM34 | Wild-XXL | XXL | 03/27/2014 | XXL-HF | 19135 |
| CDXM35 | Wild-XXL | XXL | 03/27/2014 | XXL-HP | 19931 |
| CDXM38 | Wild-XXL | XXL | 03/26/2014 | XXL-HP | 14235 |
| CDXM39 | Wild-XXL | XXL | 03/28/2014 | XXL-HP | 15320 |
| CDXM40 | Wild-XXL | XXL | 03/28/2014 | XXL-HP | 16540 |
| SHI03 | Wild-XXL | XXL | 03/13/2013 | XXL-HP | 13553 |
| SHI05 | Wild-XXL | XXL | 03/13/2013 | XXL-HP | 13857 |
| SHI06 | Wild-XXL | XXL | 03/13/2013 | XXL-HP | 9756 |
| SHI07 | Wild-XXL | XXL | 02/07/2013 | XXL-HP | 6696 |
| SHI13 | Wild-XXL | XXL | 12/09/2012 | XXL-HF | 11770 |
| SHI15 | Wild-XXL | XXL | 05/21/2013 | XXL-M | 14650 |
| A-2 | Wild-XXL | XXL | 01/17/2013 | RP-HP | 37891 |
| A-4 | Wild-XXL | XXL | 05/20/2013 | RP-HF | 30081 |
| B1-2 | Wild-XXL | XXL | 05/10/2013 | RP-HP | 37156 |
| B1-3 | Wild-XXL | XXL | 05/12/2013 | RP-M | 28549 |
| B1-4 | Wild-XXL | XXL | 11/30/2013 | RP-M | 22023 |
| B1-5 | Wild-XXL | XXL | 05/27/2013 | RP-HP | 26429 |
| B1-6 | Wild-XXL | XXL | 05/10/2013 | RP-HP | 17173 |
| B1-7 | Wild-XXL | XXL | unclear | RP-HP | 23273 |
| B2-1 | Wild-XXL | XXL | 01/12/2013 | RP-HP | 12447 |
| B2-2 | Wild-XXL | XXL | 03/28/2014 | RP-HP | 14680 |
| B2-3 | Wild-XXL | XXL | 03/28/2014 | RP-HP | 23892 |
| B2-4 | Wild-XXL | XXL | 03/28/2014 | RP-HP | 22854 |
| B2-5 | Wild-XXL | XXL | 03/16/2014 | RP-HP | 16348 |
| B2-6 | Wild-XXL | XXL | 11/03/2012 | RP-HP | 22327 |
| B2-7 | Wild-XXL | XXL | 01/22/2013 | RP-M | 17134 |
| B2-8 | Wild-XXL | XXL | 03/18/2014 | RP-HP | 35008 |
| B2-9 | Wild-XXL | XXL | 03/18/2014 | RP-HP | 15614 |
| C-1 | Wild-XXL | XXL | 03/18/2014 | RP-HP | 30789 |
| C-10 | Wild-XXL | XXL | 03/29/2014 | RP-HP | 32343 |
| C-12 | Wild-XXL | XXL | 03/17/2014 | RP-HP | 35646 |
| C-13 | Wild-XXL | XXL | 03/18/2014 | RP-M | 28725 |
| C-14 | Wild-XXL | XXL | 03/18/2014 | RP-HP | 35266 |
| C-15 | Wild-XXL | XXL | 03/16/2014 | RP-HP | 25899 |
| C-16 | Wild-XXL | XXL | 03/24/2014 | RP-HP | 28933 |
| C-17 | Wild-XXL | XXL | 03/29/2014 | RP-HP | 27507 |
| C-18 | Wild-XXL | XXL | 03/18/2014 | RP-HP | 38177 |
| C-19 | Wild-XXL | XXL | 03/28/2014 | RP-HP | 21778 |
| C-2 | Wild-XXL | XXL | 03/18/2014 | RP-HP | 35233 |
| C-20 | Wild-XXL | XXL | unclear | RP-HP | 32116 |
| C-3 | Wild-XXL | XXL | 03/18/2014 | RP-HP | 34818 |
| C-4 | Wild-XXL | XXL | 03/17/2014 | RP-M | 35431 |
| C-6 | Wild-XXL | XXL | 03/18/2014 | RP-HP | 26098 |
| C-7 | Wild-XXL | XXL | 03/29/2014 | RP-HP | 36666 |
| C-8 | Wild-XXL | XXL | 03/17/2014 | RP-HP | 28185 |
| C-9 | Wild-XXL | XXL | 03/29/2014 | RP-HP | 38647 |
| SHI10 | Wild-XXL | XXL | 05/21/2013 | RP-HF | 7503 |
| SHI11 | Wild-XXL | XXL | 06/07/2013 | RP-HP | 11043 |
| 12 | TP | ZX | Start | ZX-M | 22331 |
| 17 | TP | ZX | 03/07/2014 | ZX-HP | 21740 |
| 18 | TP | ZX | 11/29/2015 | ZX-HF | 37368 |
| 24 | TP | ZX | 03/03/2014 | ZX-HP | 39285 |
| 27 | TP | ZX | 03/16/2014 | ZX-HF | 39309 |
| 31 | TP | ZX | 10/26/2014 | ZX-HF | 23910 |
| 36 | TP | ZX | 03/14/2014 | ZX-HF | 33685 |
| 37 | TP | ZX | 05/11/2015 | ZX-HP | 24994 |
| 39 | TP | ZX | 03/02/2014 | ZX-HF | 34055 |
| 41 | TP | ZX | 03/04/2014 | ZX-M | 39146 |
| 44 | TP | ZX | 03/12/2014 | ZX-HF | 26405 |
| 47 | TP | ZX | 03/03/2014 | ZX-HP | 32641 |
| 49 | TP | ZX | 02/28/2014 | ZX-HP | 27338 |
| 51 | TP | ZX | 03/15/2014 | ZX-HF | 27220 |
| 52 | TP | ZX | 03/11/2014 | ZX-HF | 30873 |
| 55 | TP | ZX | 03/08/2014 | ZX-HP | 28241 |
| 57 | TP | ZX | 11/29/2014 | ZX-M | 17899 |
| 58 | TP | ZX | 03/01/2014 | ZX-M | 9057 |
| 1312083 | TP | ZX | 12/08/2013 | ZX-M | 18815 |
| 13111301 | TP | ZX | 11/13/2013 | ZX-HP | 19688 |
| 13111503 | TP | ZX | 11/15/2013 | ZX-M | 12786 |
| 13112003 | TP | ZX | 11/20/2013 | ZX-HF | 19207 |
| 13112203 | TP | ZX | 11/22/2013 | ZX-HF | 18143 |
| 13112403 | TP | ZX | 11/24/2013 | ZX-HP | 16390 |
| 13112503 | TP | ZX | 11/25/2013 | ZX-M | 14071 |
| 13112803 | TP | ZX | 11/28/2013 | ZX-HF | 19762 |
| 13112903 | TP | ZX | 11/29/2013 | ZX-HF | 17376 |
| 13120103 | TP | ZX | 12/01/2013 | ZX-HF | 13812 |
| 13120502 | TP | ZX | 12/05/2013 | ZX-M | 12407 |
| 13120603 | TP | ZX | 12/06/2013 | ZX-M | 15758 |
| 13120903 | TP | ZX | 12/09/2013 | ZX-M | 19748 |
| 13121102 | TP | ZX | 12/11/2013 | ZX-M | 15369 |
| 13121202 | TP | ZX | 12/12/2013 | ZX-HF | 16832 |
| 13121403 | TP | ZX | 12/14/2013 | ZX-M | 16572 |
| 13121503 | TP | ZX | 12/15/2013 | ZX-HP | 10279 |
| 13121702 | TP | ZX | 12/17/2013 | ZX-HP | 16208 |
| 13121802 | TP | ZX | 12/18/2013 | ZX-HP | 11962 |
| 13121903 | TP | ZX | 12/19/2013 | ZX-HF | 15038 |
| 13122103 | TP | ZX | 12/21/2013 | ZX-M | 15666 |
| 13122203 | TP | ZX | 12/22/2013 | ZX-M | 14924 |
| 13122403 | TP | ZX | 12/24/2013 | ZX-M | 18410 |
| 13122501 | TP | ZX | 12/25/2013 | ZX-HP | 15270 |
| 13122803 | TP | ZX | 12/28/2013 | ZX-M | 11270 |
| 14010602 | TP | ZX | 01/06/2014 | ZX-HF | 12458 |
| 14010701 | TP | ZX | 01/07/2014 | ZX-HF | 12184 |
| 14010903 | TP | ZX | 01/09/2014 | ZX-HF | 16004 |
| 14011103 | TP | ZX | 01/11/2014 | ZX-HF | 17413 |
| 14011203 | TP | ZX | 01/12/2014 | ZX-HF | 11649 |
| 14011403 | TP | ZX | 01/14/2014 | ZX-HF | 19807 |
| 14011603 | TP | ZX | 01/16/2014 | ZX-M | 14054 |
| 14011802 | TP | ZX | 01/18/2014 | ZX-HF | 12448 |
| 14012003 | TP | ZX | 01/20/2014 | ZX-HF | 16651 |
| 14012103 | TP | ZX | 01/21/2014 | ZX-HF | 15477 |
| 14012302 | TP | ZX | 01/23/2014 | ZX-M | 13433 |
| 14012403 | TP | ZX | 01/24/2014 | ZX-HF | 15047 |
| 14012603 | TP | ZX | 01/26/2014 | ZX-HF | 11897 |
| 14012803 | TP | ZX | 01/28/2014 | ZX-HP | 16087 |
| 14021003 | TP | ZX | 02/10/2014 | ZX-M | 16038 |
| 14021103 | TP | ZX | 02/11/2014 | ZX-HF | 11888 |
| 14021403 | TP | ZX | 02/14/2014 | ZX-M | 19341 |
| 14021503 | TP | ZX | 02/15/2014 | ZX-HF | 13830 |
| 14021803 | TP | ZX | 02/18/2014 | ZX-HF | 13940 |
| 20131126 | TP | ZX | 11/26/2013 | ZX-M | 15467 |
| 20131203 | TP | ZX | 12/03/2013 | ZX-M | 19312 |
| 20131210 | TP | ZX | 12/10/2013 | ZX-M | 13638 |
| 20131216 | TP | ZX | 12/16/2013 | ZX-M | 17619 |
| 20131223 | TP | ZX | 12/23/2013 | ZX-M | 19865 |
| 20140113 | TP | ZX | 01/13/2014 | ZX-HF | 9505 |
| 20140117 | TP | ZX | 01/17/2014 | ZX-M | 17236 |
| 20140122 | TP | ZX | 01/22/2014 | ZX-HF | 17773 |
| 20140127 | TP | ZX | 01/27/2014 | ZX-HP | 17474 |
| 20140213 | TP | ZX | 02/13/2014 | ZX-HF | 11151 |
| 0105F01 | TP | ZX | 01/05/2014 | ZX-HF | 24716 |
| 0108F01 | TP | ZX | 01/08/2014 | ZX-HF | 24742 |
| 0115F03 | TP | ZX | 01/15/2014 | ZX-HF | 25417 |
| 0119F04 | TP | ZX | 01/19/2014 | ZX-HF | 28603 |
| 0125F03 | TP | ZX | 01/25/2014 | ZX-M | 14008 |
| 0209F03 | TP | ZX | 02/09/2014 | ZX-M | 23462 |
| 0216F03 | TP | ZX | 02/16/2014 | ZX-HF | 18945 |
| 0221F03 | TP | ZX | 02/21/2014 | ZX-HF | 16910 |
| 1112F03 | TP | ZX | 11/12/2013 | ZX-M | 23303 |
| 1114F03 | TP | ZX | 11/14/2013 | ZX-M | 27291 |
| 1116F03 | TP | ZX | 11/16/2013 | ZX-HP | 19771 |
| 1123F03 | TP | ZX | 11/23/2013 | ZX-HF | 24203 |
| 1130F03 | TP | ZX | 11/30/2013 | ZX-M | 22234 |
| 11zl | TP | ZX | 12/08/2015 | ZX-M | 29654 |
| 1207F03 | TP | ZX | 12/07/2013 | ZX-HF | 18228 |
| 1213F03 | TP | ZX | 12/13/2013 | ZX-M | 22207 |
| 1220F03 | TP | ZX | 12/20/2013 | ZX-HF | 23013 |
| 1227F02 | TP | ZX | 12/27/2013 | ZX-HP | 10301 |
| 1229F03 | TP | ZX | 12/29/2013 | ZX-HP | 27737 |
| 1zl | TP | ZX | 11/29/2014 | ZX-HF | 36513 |
| 8zl | TP | ZX | 03/06/2014 | ZX-HP | 39466 |
| CDXM01 | TP | ZX | 04/30/2014 | ZX-HP | 12017 |
| CDXM02 | TP | ZX | 02/24/2014 | ZX-HF | 10356 |
| CDXM03 | TP | ZX | 02/27/2014 | ZX-HF | 11723 |
| CDXM04 | TP | ZX | 03/29/2014 | ZX-M | 9992 |
| CDXM05 | TP | ZX | 03/28/2014 | ZX-HF | 15684 |
| CDXM07 | TP | ZX | 02/25/2014 | ZX-HF | 11089 |
| CDXM08 | TP | ZX | 03/26/2014 | ZX-HF | 11212 |
| CDXM09 | TP | ZX | 02/23/2014 | ZX-HF | 13139 |
| CDXM10 | TP | ZX | 02/22/2014 | ZX-HF | 14132 |
| CDXM11 | TP | ZX | 02/26/2014 | ZX-HF | 15009 |
| CDXM12 | TP | ZX | 04/19/2014 | ZX-M | 15067 |
| CDXM13 | TP | ZX | 03/25/2014 | ZX-HF | 15661 |
| CDXM14 | TP | ZX | 03/27/2014 | ZX-M | 14127 |
| CDXM16 | TP | ZX | 05/27/2014 | ZX-HF | 15698 |

TP, captive-origin translocated giant pandas; Wild, meaning samples from wild populations; MS, Minshan Mountain population; RP, red pandas; XXL, Xiaoxiangling Mountain population. HF, high proportion of Firmicutes; HP, high proportion of Proteobacteria. XXLuncl, the sample information was not clear.

**Table S2** Detailed information (collection time and groups) of Père David's deer (*Elaphurus davidianus*) fecal samples*

| Sample ID | Collecting time | Group1 | Region | Group | Clean-read |
| --- | --- | --- | --- | --- | --- |
| 5 | 2012.12.28 | 11-12.DF.winter.core2 | DF2 | DF | 9617 |
| 23 | 2012.2.18 | 11-12.DF.winter.core3 | DF3 | DF | 6762 |
| 42 | 2012.1.16 | 11-12.DF.winter.core3 | DF3 | DF | 7266 |
| 73 | 2011.11.28 | 11-12.DF.winter.core2 | DF2 | DF | 9540 |
| 74 | 2011.11.28 | 11-12.DF.winter.core2 | DF2 | DF | 8624 |
| 75 | 2011.11.28 | 11-12.DF.winter.core2 | DF2 | DF | 9663 |
| 76 | 2011.11.28 | 11-12.DF.winter.core2 | DF2 | DF | 10350 |
| 77 | 2011.11.28 | 11-12.DF.winter.core2 | DF2 | DF | 11362 |
| 78 | 2011.11.28 | 11-12.DF.winter.core2 | DF2 | DF | 8998 |
| 111 | 2012.1.17 | 11-12.DF.winter.core3 | DF3 | DF | 6868 |
| 018M2 | 2011.11.20 | 11-12.DF.winter.core1 | DF1 | DF | 8500 |
| DF049 | 2012.1.16 | 11-12.DF.winter.core3 | DF3 | DF | 9481 |
| DF137 | 2012.1.17 | 11-12.DF.winter.core3 | DF3 | DF | 5364 |
| DF2.1 | 2014.9.25 | 14.DF.summer.core2 | DF2 | DF | 6031 |
| DF2.10 | 2014.9.25 | 14.DF.summer.core2 | DF2 | DF | 8242 |
| DF2.11 | 2014.9.25 | 14.DF.summer.core2 | DF2 | DF | 9880 |
| DF2.14 | 2014.9.25 | 14.DF.summer.core2 | DF2 | DF | 7589 |
| DF2.15 | 2014.9.25 | 14.DF.summer.core2 | DF2 | DF | 8778 |
| DF2.16 | 2014.9.25 | 14.DF.summer.core2 | DF2 | DF | 7634 |
| DF2.17 | 2014.9.25 | 14.DF.summer.core2 | DF2 | DF | 7783 |
| DF2.2 | 2014.9.25 | 14.DF.summer.core2 | DF2 | DF | 6435 |
| DF2.20 | 2014.9.25 | 14.DF.summer.core2 | DF2 | DF | 6588 |
| DF2.22 | 2014.9.25 | 14.DF.summer.core2 | DF2 | DF | 11863 |
| DF2.23 | 2014.9.25 | 14.DF.summer.core2 | DF2 | DF | 7566 |
| DF2.24 | 2014.9.25 | 14.DF.summer.core2 | DF2 | DF | 10442 |
| DF2.25 | 2014.9.25 | 14.DF.summer.core2 | DF2 | DF | 8103 |
| DF2.26 | 2014.9.25 | 14.DF.summer.core2 | DF2 | DF | 7877 |
| DF2.3 | 2014.9.25 | 14.DF.summer.core2 | DF2 | DF | 6617 |
| DF2.4 | 2014.9.25 | 14.DF.summer.core2 | DF2 | DF | 9456 |
| DF2.5 | 2014.9.25 | 14.DF.summer.core2 | DF2 | DF | 7966 |
| DF2.6 | 2014.9.25 | 14.DF.summer.core2 | DF2 | DF | 4794 |
| DF2.7 | 2014.9.25 | 14.DF.summer.core2 | DF2 | DF | 5702 |
| DF2.8 | 2014.9.25 | 14.DF.summer.core2 | DF2 | DF | 7378 |
| DF3.1 | 2014.9.26 | 14.DF.summer.core3 | DF3 | DF | 9619 |
| DF3.10 | 2014.9.26 | 14.DF.summer.core3 | DF3 | DF | 7222 |
| DF3.11 | 2014.9.26 | 14.DF.summer.core3 | DF3 | DF | 9570 |
| DF3.12 | 2014.9.26 | 14.DF.summer.core3 | DF3 | DF | 6304 |
| DF3.13 | 2014.9.26 | 14.DF.summer.core3 | DF3 | DF | 7901 |
| DF3.14 | 2014.9.26 | 14.DF.summer.core3 | DF3 | DF | 4989 |
| DF3.15 | 2014.9.26 | 14.DF.summer.core3 | DF3 | DF | 6805 |
| DF3.16 | 2014.9.26 | 14.DF.summer.core3 | DF3 | DF | 7443 |
| DF3.17 | 2014.9.26 | 14.DF.summer.core3 | DF3 | DF | 7683 |
| DF3.18 | 2014.9.26 | 14.DF.summer.core3 | DF3 | DF | 9017 |
| DF3.19 | 2014.9.26 | 14.DF.summer.core3 | DF3 | DF | 6405 |
| DF3.2 | 2014.9.26 | 14.DF.summer.core3 | DF3 | DF | 6685 |
| DF3.20 | 2014.9.26 | 14.DF.summer.core3 | DF3 | DF | 5231 |
| DF3.21 | 2014.9.26 | 14.DF.summer.core3 | DF3 | DF | 5047 |
| DF3.22 | 2014.9.26 | 14.DF.summer.core3 | DF3 | DF | 8918 |
| DF3.23 | 2014.9.26 | 14.DF.summer.core3 | DF3 | DF | 6668 |
| DF3.24 | 2014.9.26 | 14.DF.summer.core3 | DF3 | DF | 6409 |
| DF3.25 | 2014.9.26 | 14.DF.summer.core3 | DF3 | DF | 8760 |
| DF3.26 | 2014.9.26 | 14.DF.summer.core3 | DF3 | DF | 9916 |
| DF3.27 | 2014.9.26 | 14.DF.summer.core3 | DF3 | DF | 7810 |
| DF3.28 | 2014.9.26 | 14.DF.summer.core3 | DF3 | DF | 6334 |
| DF3.29 | 2014.9.26 | 14.DF.summer.core3 | DF3 | DF | 7251 |
| DF3.3 | 2014.9.26 | 14.DF.summer.core3 | DF3 | DF | 10186 |
| DF3.30 | 2014.9.26 | 14.DF.summer.core3 | DF3 | DF | 8910 |
| DF3.32 | 2014.9.26 | 14.DF.summer.core3 | DF3 | DF | 10060 |
| DF3.4 | 2014.9.26 | 14.DF.summer.core3 | DF3 | DF | 7132 |
| DFW1.1 | 2014.11.26 | 14.DF.winter.core1 | DF1 | DF | 6716 |
| DFW1.10 | 2014.11.26 | 14.DF.winter.core1 | DF1 | DF | 8266 |
| DFW1.11 | 2014.11.26 | 14.DF.winter.core1 | DF1 | DF | 7953 |
| DFW1.12 | 2014.11.26 | 14.DF.winter.core1 | DF1 | DF | 7235 |
| DFW1.13 | 2014.11.26 | 14.DF.winter.core1 | DF1 | DF | 9385 |
| DFW1.14 | 2014.11.26 | 14.DF.winter.core1 | DF1 | DF | 7557 |
| DFW1.15 | 2014.11.26 | 14.DF.winter.core1 | DF1 | DF | 7746 |
| DFW1.16 | 2014.11.26 | 14.DF.winter.core1 | DF1 | DF | 9020 |
| DFW1.17 | 2014.11.26 | 14.DF.winter.core1 | DF1 | DF | 9382 |
| DFW1.18 | 2014.11.26 | 14.DF.winter.core1 | DF1 | DF | 5487 |
| DFW1.19 | 2014.11.26 | 14.DF.winter.core1 | DF1 | DF | 9829 |
| DFW1.2 | 2014.11.26 | 14.DF.winter.core1 | DF1 | DF | 10406 |
| DFW1.20 | 2014.11.26 | 14.DF.winter.core1 | DF1 | DF | 4891 |
| DFW1.21 | 2014.11.26 | 14.DF.winter.core1 | DF1 | DF | 8874 |
| DFW1.22 | 2014.11.26 | 14.DF.winter.core1 | DF1 | DF | 7795 |
| DFW1.23 | 2014.11.26 | 14.DF.winter.core1 | DF1 | DF | 5096 |
| DFW1.24 | 2014.11.26 | 14.DF.winter.core1 | DF1 | DF | 8709 |
| DFW1.25 | 2014.11.26 | 14.DF.winter.core1 | DF1 | DF | 9390 |
| DFW1.26 | 2014.11.26 | 14.DF.winter.core1 | DF1 | DF | 11170 |
| DFW1.27 | 2014.11.26 | 14.DF.winter.core1 | DF1 | DF | 12363 |
| DFW1.28 | 2014.11.26 | 14.DF.winter.core1 | DF1 | DF | 11722 |
| DFW1.29 | 2014.11.26 | 14.DF.winter.core1 | DF1 | DF | 9082 |
| DFW1.3 | 2014.11.26 | 14.DF.winter.core1 | DF1 | DF | 14310 |
| DFW1.30 | 2014.11.26 | 14.DF.winter.core1 | DF1 | DF | 10251 |
| DFW1.31 | 2014.11.26 | 14.DF.winter.core1 | DF1 | DF | 10262 |
| DFW1.32 | 2014.11.26 | 14.DF.winter.core1 | DF1 | DF | 9197 |
| DFW1.33 | 2014.11.26 | 14.DF.winter.core1 | DF1 | DF | 9484 |
| DFW1.34 | 2014.11.26 | 14.DF.winter.core1 | DF1 | DF | 8360 |
| DFW1.35 | 2014.11.26 | 14.DF.winter.core1 | DF1 | DF | 9047 |
| DFW1.36 | 2014.11.26 | 14.DF.winter.core1 | DF1 | DF | 6322 |
| DFW1.4 | 2014.11.26 | 14.DF.winter.core1 | DF1 | DF | 9639 |
| DFW1.5 | 2014.11.26 | 14.DF.winter.core1 | DF1 | DF | 9128 |
| DFW1.6 | 2014.11.26 | 14.DF.winter.core1 | DF1 | DF | 9068 |
| DFW1.7 | 2014.11.26 | 14.DF.winter.core1 | DF1 | DF | 9778 |
| DFW1.8 | 2014.11.26 | 14.DF.winter.core1 | DF1 | DF | 8129 |
| DFW1.9 | 2014.11.26 | 14.DF.winter.core1 | DF1 | DF | 7569 |
| DFW2.1 | 2014.11.26 | 14.DF.winter.core2 | DF2 | DF | 8992 |
| DFW2.10 | 2014.11.26 | 14.DF.winter.core2 | DF2 | DF | 7039 |
| DFW2.11 | 2014.11.26 | 14.DF.winter.core2 | DF2 | DF | 6133 |
| DFW2.12 | 2014.11.26 | 14.DF.winter.core2 | DF2 | DF | 6706 |
| DFW2.13 | 2014.11.26 | 14.DF.winter.core2 | DF2 | DF | 6540 |
| DFW2.14 | 2014.11.26 | 14.DF.winter.core2 | DF2 | DF | 8249 |
| DFW2.15 | 2014.11.26 | 14.DF.winter.core2 | DF2 | DF | 8374 |
| DFW2.16 | 2014.11.26 | 14.DF.winter.core2 | DF2 | DF | 6593 |
| DFW2.17 | 2014.11.26 | 14.DF.winter.core2 | DF2 | DF | 7817 |
| DFW2.18 | 2014.11.26 | 14.DF.winter.core2 | DF2 | DF | 8681 |
| DFW2.19 | 2014.11.26 | 14.DF.winter.core2 | DF2 | DF | 4508 |
| DFW2.2 | 2014.11.26 | 14.DF.winter.core2 | DF2 | DF | 7452 |
| DFW2.20 | 2014.11.26 | 14.DF.winter.core2 | DF2 | DF | 8181 |
| DFW2.21 | 2014.11.26 | 14.DF.winter.core2 | DF2 | DF | 10979 |
| DFW2.22 | 2014.11.26 | 14.DF.winter.core2 | DF2 | DF | 8028 |
| DFW2.23 | 2014.11.26 | 14.DF.winter.core2 | DF2 | DF | 5452 |
| DFW2.24 | 2014.11.26 | 14.DF.winter.core2 | DF2 | DF | 4959 |
| DFW2.25 | 2014.11.26 | 14.DF.winter.core2 | DF2 | DF | 7177 |
| DFW2.26 | 2014.11.26 | 14.DF.winter.core2 | DF2 | DF | 7627 |
| DFW2.27 | 2014.11.26 | 14.DF.winter.core2 | DF2 | DF | 6287 |
| DFW2.28 | 2014.11.26 | 14.DF.winter.core2 | DF2 | DF | 6237 |
| DFW2.29 | 2014.11.26 | 14.DF.winter.core2 | DF2 | DF | 7800 |
| DFW2.3 | 2014.11.26 | 14.DF.winter.core2 | DF2 | DF | 9353 |
| DFW2.30 | 2014.11.26 | 14.DF.winter.core2 | DF2 | DF | 9699 |
| DFW2.31 | 2014.11.26 | 14.DF.winter.core2 | DF2 | DF | 7413 |
| DFW2.32 | 2014.11.26 | 14.DF.winter.core2 | DF2 | DF | 8745 |
| DFW2.33 | 2014.11.26 | 14.DF.winter.core2 | DF2 | DF | 8379 |
| DFW2.34 | 2014.11.26 | 14.DF.winter.core2 | DF2 | DF | 7086 |
| DFW2.35 | 2014.11.26 | 14.DF.winter.core2 | DF2 | DF | 6616 |
| DFW2.36 | 2014.11.26 | 14.DF.winter.core2 | DF2 | DF | 8535 |
| DFW2.37 | 2014.11.26 | 14.DF.winter.core2 | DF2 | DF | 7968 |
| DFW2.38 | 2014.11.26 | 14.DF.winter.core2 | DF2 | DF | 6553 |
| DFW2.39 | 2014.11.26 | 14.DF.winter.core2 | DF2 | DF | 9563 |
| DFW2.4 | 2014.11.26 | 14.DF.winter.core2 | DF2 | DF | 8611 |
| DFW2.40 | 2014.11.26 | 14.DF.winter.core2 | DF2 | DF | 5350 |
| DFW2.41 | 2014.11.26 | 14.DF.winter.core2 | DF2 | DF | 6457 |
| DFW2.5 | 2014.11.26 | 14.DF.winter.core2 | DF2 | DF | 8805 |
| DFW2.6 | 2014.11.26 | 14.DF.winter.core2 | DF2 | DF | 9284 |
| DFW2.7 | 2014.11.26 | 14.DF.winter.core2 | DF2 | DF | 8859 |
| DFW2.8 | 2014.11.26 | 14.DF.winter.core2 | DF2 | DF | 6249 |
| DFW2.9 | 2014.11.26 | 14.DF.winter.core2 | DF2 | DF | 8823 |
| DFW3.1 | 2014.11.27 | 14.DF.winter.core3 | DF3 | DF | 7694 |
| DFW3.10 | 2014.11.27 | 14.DF.winter.core3 | DF3 | DF | 6868 |
| DFW3.11 | 2014.11.27 | 14.DF.winter.core3 | DF3 | DF | 6213 |
| DFW3.12 | 2014.11.27 | 14.DF.winter.core3 | DF3 | DF | 7523 |
| DFW3.13 | 2014.11.27 | 14.DF.winter.core3 | DF3 | DF | 8163 |
| DFW3.14 | 2014.11.27 | 14.DF.winter.core3 | DF3 | DF | 7115 |
| DFW3.15 | 2014.11.27 | 14.DF.winter.core3 | DF3 | DF | 7254 |
| DFW3.16 | 2014.11.27 | 14.DF.winter.core3 | DF3 | DF | 9846 |
| DFW3.17 | 2014.11.27 | 14.DF.winter.core3 | DF3 | DF | 5081 |
| DFW3.18 | 2014.11.27 | 14.DF.winter.core3 | DF3 | DF | 7755 |
| DFW3.19 | 2014.11.27 | 14.DF.winter.core3 | DF3 | DF | 7943 |
| DFW3.2 | 2014.11.27 | 14.DF.winter.core3 | DF3 | DF | 10664 |
| DFW3.20 | 2014.11.27 | 14.DF.winter.core3 | DF3 | DF | 6187 |
| DFW3.21 | 2014.11.27 | 14.DF.winter.core3 | DF3 | DF | 7723 |
| DFW3.22 | 2014.11.27 | 14.DF.winter.core3 | DF3 | DF | 4649 |
| DFW3.23 | 2014.11.27 | 14.DF.winter.core3 | DF3 | DF | 7433 |
| DFW3.24 | 2014.11.27 | 14.DF.winter.core3 | DF3 | DF | 7351 |
| DFW3.25 | 2014.11.27 | 14.DF.winter.core3 | DF3 | DF | 8154 |
| DFW3.26 | 2014.11.27 | 14.DF.winter.core3 | DF3 | DF | 6901 |
| DFW3.27 | 2014.11.27 | 14.DF.winter.core3 | DF3 | DF | 4811 |
| DFW3.28 | 2014.11.27 | 14.DF.winter.core3 | DF3 | DF | 4960 |
| DFW3.29 | 2014.11.27 | 14.DF.winter.core3 | DF3 | DF | 8299 |
| DFW3.3 | 2014.11.27 | 14.DF.winter.core3 | DF3 | DF | 7109 |
| DFW3.30 | 2014.11.27 | 14.DF.winter.core3 | DF3 | DF | 7113 |
| DFW3.31 | 2014.11.27 | 14.DF.winter.core3 | DF3 | DF | 8168 |
| DFW3.32 | 2014.11.27 | 14.DF.winter.core3 | DF3 | DF | 7361 |
| DFW3.33 | 2014.11.27 | 14.DF.winter.core3 | DF3 | DF | 5422 |
| DFW3.34 | 2014.11.27 | 14.DF.winter.core3 | DF3 | DF | 4579 |
| DFW3.35 | 2014.11.27 | 14.DF.winter.core3 | DF3 | DF | 5520 |
| DFW3.36 | 2014.11.27 | 14.DF.winter.core3 | DF3 | DF | 8643 |
| DFW3.37 | 2014.11.27 | 14.DF.winter.core3 | DF3 | DF | 8263 |
| DFW3.38 | 2014.11.27 | 14.DF.winter.core3 | DF3 | DF | 6607 |
| DFW3.39 | 2014.11.27 | 14.DF.winter.core3 | DF3 | DF | 7590 |
| DFW3.4 | 2014.11.27 | 14.DF.winter.core3 | DF3 | DF | 8388 |
| DFW3.40 | 2014.11.27 | 14.DF.winter.core3 | DF3 | DF | 9430 |
| DFW3.41 | 2014.11.27 | 14.DF.winter.core3 | DF3 | DF | 7284 |
| DFW3.42 | 2014.11.27 | 14.DF.winter.core3 | DF3 | DF | 5276 |
| DFW3.43 | 2014.11.27 | 14.DF.winter.core3 | DF3 | DF | 5069 |
| DFW3.44 | 2014.11.27 | 14.DF.winter.core3 | DF3 | DF | 5374 |
| DFW3.45 | 2014.11.27 | 14.DF.winter.core3 | DF3 | DF | 9831 |
| DFW3.46 | 2014.11.27 | 14.DF.winter.core3 | DF3 | DF | 10015 |
| DFW3.47 | 2014.11.27 | 14.DF.winter.core3 | DF3 | DF | 4509 |
| DFW3.48 | 2014.11.27 | 14.DF.winter.core3 | DF3 | DF | 6985 |
| DFW3.5 | 2014.11.27 | 14.DF.winter.core3 | DF3 | DF | 5307 |
| DFW3.6 | 2014.11.27 | 14.DF.winter.core3 | DF3 | DF | 6967 |
| DFW3.7 | 2014.11.27 | 14.DF.winter.core3 | DF3 | DF | 8257 |
| DFW3.8 | 2014.11.27 | 14.DF.winter.core3 | DF3 | DF | 9224 |
| DFW3.9 | 2014.11.27 | 14.DF.winter.core3 | DF3 | DF | 6733 |
| ED1.1 | 2014.9.25 | 14.DF.summer.core1 | DF1 | DF | 8677 |
| ED1.10 | 2014.9.25 | 14.DF.summer.core1 | DF1 | DF | 8575 |
| ED1.11 | 2014.9.25 | 14.DF.summer.core1 | DF1 | DF | 8954 |
| ED1.12 | 2014.9.25 | 14.DF.summer.core1 | DF1 | DF | 10817 |
| ED1.13 | 2014.9.25 | 14.DF.summer.core1 | DF1 | DF | 7817 |
| ED1.14 | 2014.9.25 | 14.DF.summer.core1 | DF1 | DF | 9275 |
| ED1.15 | 2014.9.25 | 14.DF.summer.core1 | DF1 | DF | 10377 |
| ED1.16 | 2014.9.25 | 14.DF.summer.core1 | DF1 | DF | 6588 |
| ED1.17 | 2014.9.25 | 14.DF.summer.core1 | DF1 | DF | 5042 |
| ED1.18 | 2014.9.25 | 14.DF.summer.core1 | DF1 | DF | 10107 |
| ED1.19 | 2014.9.25 | 14.DF.summer.core1 | DF1 | DF | 8911 |
| ED1.2 | 2014.9.25 | 14.DF.summer.core1 | DF1 | DF | 6847 |
| ED1.20 | 2014.9.25 | 14.DF.summer.core1 | DF1 | DF | 9193 |
| ED1.21 | 2014.9.25 | 14.DF.summer.core1 | DF1 | DF | 7511 |
| ED1.23 | 2014.9.25 | 14.DF.summer.core1 | DF1 | DF | 9135 |
| ED1.24 | 2014.9.25 | 14.DF.summer.core1 | DF1 | DF | 8620 |
| ED1.25 | 2014.9.25 | 14.DF.summer.core1 | DF1 | DF | 10174 |
| ED1.26 | 2014.9.25 | 14.DF.summer.core1 | DF1 | DF | 7983 |
| ED1.3 | 2014.9.25 | 14.DF.summer.core1 | DF1 | DF | 8586 |
| ED1.4 | 2014.9.25 | 14.DF.summer.core1 | DF1 | DF | 9270 |
| ED1.5 | 2014.9.25 | 14.DF.summer.core1 | DF1 | DF | 8929 |
| ED1.6 | 2014.9.25 | 14.DF.summer.core1 | DF1 | DF | 7011 |
| ED1.7 | 2014.9.25 | 14.DF.summer.core1 | DF1 | DF | 9388 |
| ED1.8 | 2014.9.25 | 14.DF.summer.core1 | DF1 | DF | 5617 |
| ED1.9 | 2014.9.25 | 14.DF.summer.core1 | DF1 | DF | 7166 |
| M1 | 2011.11.20 | 11-12.DF.winter.core3 | DF3 | DF | 7114 |
| M3 | 2011.11.20 | 11-12.DF.winter.core2 | DF2 | DF | 6346 |
| ML1.5 | 2013.8.26 | 13.DF.summer.core1 | DF1 | DF | 9152 |
| ML1.6 | 2013.8.26 | 13.DF.summer.core1 | DF1 | DF | 8052 |
| ML1.7 | 2013.8.26 | 13.DF.summer.core1 | DF1 | DF | 13602 |
| ML1.8 | 2013.8.26 | 13.DF.summer.core1 | DF1 | DF | 10733 |
| ML1.9 | 2013.8.26 | 13.DF.summer.core1 | DF1 | DF | 9275 |
| ML2.10 | 2013.8.26 | 13.DF.summer.core2 | DF2 | DF | 10193 |
| ML2.11 | 2013.8.26 | 13.DF.summer.core2 | DF2 | DF | 12618 |
| ML2.12 | 2013.8.26 | 13.DF.summer.core2 | DF2 | DF | 11891 |
| ML2.13 | 2013.8.26 | 13.DF.summer.core2 | DF2 | DF | 9693 |
| ML2.4 | 2013.8.26 | 13.DF.summer.core2 | DF2 | DF | 12928 |
| ML2.5 | 2013.8.26 | 13.DF.summer.core2 | DF2 | DF | 15072 |
| ML2.6 | 2013.8.26 | 13.DF.summer.core2 | DF2 | DF | 10951 |
| ML2.7 | 2013.8.26 | 13.DF.summer.core2 | DF2 | DF | 11705 |
| ML2.8 | 2013.8.26 | 13.DF.summer.core2 | DF2 | DF | 15679 |
| ML3.1 | 2013.8.27 | 13.DF.summer.core3 | DF3 | DF | 14911 |
| ML3.2 | 2013.8.27 | 13.DF.summer.core3 | DF3 | DF | 13036 |
| ML3.21 | 2013.8.27 | 13.DF.summer.core3 | DF3 | DF | 12597 |
| ML3.22 | 2013.8.27 | 13.DF.summer.core3 | DF3 | DF | 15143 |
| ML3.23 | 2013.8.27 | 13.DF.summer.core3 | DF3 | DF | 11765 |
| ML3.3 | 2013.8.27 | 13.DF.summer.core3 | DF3 | DF | 15045 |
| ML3.5 | 2013.8.27 | 13.DF.summer.core3 | DF3 | DF | 14479 |
| ML3.6 | 2013.8.27 | 13.DF.summer.core3 | DF3 | DF | 12822 |
| ML3.7 | 2013.8.27 | 13.DF.summer.core3 | DF3 | DF | 9735 |
| ML3.9 | 2013.8.27 | 13.DF.summer.core3 | DF3 | DF | 15249 |
| ML71 | 2012.1.16 | 11-12.DF.winter.core3 | DF3 | DF | 8475 |
| S22 | 2011.11.20 | 11-12.DF.winter.core2 | DF2 | DF | 7631 |
| S23 | 2011.11.20 | 11-12.DF.winter.core2 | DF2 | DF | 9572 |
| S24 | 2011.11.20 | 11-12.DF.winter.core2 | DF2 | DF | 7979 |
| S25 | 2011.11.20 | 11-12.DF.winter.core1 | DF1 | DF | 9934 |
| S26 | 2011.11.20 | 11-12.DF.winter.core1 | DF1 | DF | 9007 |
| S27 | 2011.11.20 | 11-12.DF.winter.core3 | DF3 | DF | 6896 |
| S28 | 2011.11.20 | 11-12.DF.winter.core3 | DF3 | DF | 7692 |
| S29 | 2011.11.20 | 11-12.DF.winter.core1 | DF1 | DF | 7757 |
| S30 | 2011.11.20 | 11-12.DF.winter.core3 | DF3 | DF | 5604 |
| SH.1 | 2014.11.5 | 14.HB.winter | HB | SH | 6972 |
| SH.10 | 2014.11.5 | 14.HB.winter | HB | SH | 9342 |
| SH.11 | 2014.11.5 | 14.HB.winter | HB | SH | 7716 |
| SH.12 | 2014.11.5 | 14.HB.winter | HB | SH | 7065 |
| SH.13 | 2014.11.5 | 14.HB.winter | HB | SH | 9631 |
| SH.14 | 2014.11.5 | 14.HB.winter | HB | SH | 8399 |
| SH.15 | 2014.11.5 | 14.HB.winter | HB | SH | 8200 |
| SH.16 | 2014.11.5 | 14.HB.winter | HB | SH | 9510 |
| SH.17 | 2014.11.5 | 14.HB.winter | HB | SH | 7148 |
| SH.18 | 2014.11.5 | 14.HB.winter | HB | SH | 7000 |
| SH.19 | 2014.11.5 | 14.HB.winter | HB | SH | 9245 |
| SH.2 | 2014.11.5 | 14.HB.winter | HB | SH | 9751 |
| SH.20 | 2014.11.5 | 14.HB.winter | HB | SH | 9064 |
| SH.21 | 2014.11.5 | 14.HB.winter | HB | SH | 9596 |
| SH.22 | 2014.11.5 | 14.HB.winter | HB | SH | 11336 |
| SH.23 | 2014.11.5 | 14.HB.winter | HB | SH | 7901 |
| SH.24 | 2014.11.5 | 14.HB.winter | HB | SH | 8948 |
| SH.25 | 2014.11.5 | 14.HB.winter | HB | SH | 8466 |
| SH.26 | 2014.11.5 | 14.HB.winter | HB | SH | 7509 |
| SH.27 | 2014.11.5 | 14.HB.winter | HB | SH | 8449 |
| SH.28 | 2014.11.5 | 14.HB.winter | HB | SH | 8264 |
| SH.29 | 2014.11.5 | 14.HB.winter | HB | SH | 9327 |
| SH.3 | 2014.11.5 | 14.HB.winter | HB | SH | 7831 |
| SH.30 | 2014.11.5 | 14.HB.winter | HB | SH | 9598 |
| SH.31 | 2014.11.5 | 14.HB.winter | HB | SH | 6217 |
| SH.32 | 2014.11.5 | 14.HB.winter | HB | SH | 4236 |
| SH.33 | 2014.11.5 | 14.HB.winter | HB | SH | 10248 |
| SH.34 | 2014.11.5 | 14.HB.winter | HB | SH | 11039 |
| SH.35 | 2014.11.5 | 14.HB.winter | HB | SH | 7932 |
| SH.36 | 2014.11.5 | 14.HB.winter | HB | SH | 10665 |
| SH.37 | 2014.11.5 | 14.HB.winter | HB | SH | 9620 |
| SH.38 | 2014.11.5 | 14.HB.winter | HB | SH | 10420 |
| SH.39 | 2014.11.5 | 14.HB.winter | HB | SH | 8524 |
| SH.4 | 2014.11.5 | 14.HB.winter | HB | SH | 9040 |
| SH.40 | 2014.11.5 | 14.HB.winter | HB | SH | 9838 |
| SH.41 | 2014.11.5 | 14.HB.winter | HB | SH | 10595 |
| SH.42 | 2014.11.5 | 14.HB.winter | HB | SH | 8468 |
| SH.43 | 2014.11.5 | 14.HB.winter | HB | SH | 9580 |
| SH.44 | 2014.11.5 | 14.HB.winter | HB | SH | 8454 |
| SH.45 | 2014.11.5 | 14.HB.winter | HB | SH | 10096 |
| SH.48 | 2014.11.5 | 14.HB.winter | HB | SH | 10572 |
| SH.49 | 2014.11.5 | 14.HB.winter | HB | SH | 13726 |
| SH.5 | 2014.11.5 | 14.HB.winter | HB | SH | 9039 |
| SH.50 | 2014.11.5 | 14.HB.winter | HB | SH | 11736 |
| SH.51 | 2014.11.5 | 14.HB.winter | HB | SH | 9983 |
| SH.52 | 2014.11.5 | 14.HB.winter | HB | SH | 11072 |
| SH.53 | 2014.11.5 | 14.HB.winter | HB | SH | 8470 |
| SH.54 | 2014.11.5 | 14.HB.winter | HB | SH | 9059 |
| SH.55 | 2014.11.5 | 14.HB.winter | HB | SH | 8130 |
| SH.56 | 2014.11.5 | 14.HB.winter | HB | SH | 9020 |
| SH.57 | 2014.11.5 | 14.HB.winter | HB | SH | 12027 |
| SH.58 | 2014.11.5 | 14.HB.winter | HB | SH | 7898 |
| SH.59 | 2014.11.5 | 14.HB.winter | HB | SH | 11150 |
| SH.6 | 2014.11.5 | 14.HB.winter | HB | SH | 7737 |
| SH.60 | 2014.11.5 | 14.HB.winter | HB | SH | 10061 |
| SH.61 | 2014.11.5 | 14.HB.winter | HB | SH | 9344 |
| SH.62 | 2014.11.5 | 14.HB.winter | HB | SH | 11448 |
| SH.63 | 2014.11.5 | 14.HB.winter | HB | SH | 11270 |
| SH.64 | 2014.11.5 | 14.HB.winter | HB | SH | 10373 |
| SH.66 | 2014.11.5 | 14.HB.winter | HB | SH | 9741 |
| SH.67 | 2014.11.5 | 14.HB.winter | HB | SH | 12096 |
| SH.68 | 2014.11.5 | 14.HB.winter | HB | SH | 10840 |
| SH.69 | 2014.11.5 | 14.HB.winter | HB | SH | 9716 |
| SH.7 | 2014.11.5 | 14.HB.winter | HB | SH | 10696 |
| SH.70 | 2014.11.5 | 14.HB.winter | HB | SH | 9628 |
| SH.71 | 2014.11.5 | 14.HB.winter | HB | SH | 10017 |
| SH.72 | 2014.11.5 | 14.HB.winter | HB | SH | 8723 |
| SH.73 | 2014.11.5 | 14.HB.winter | HB | SH | 7392 |
| SH.8 | 2014.11.5 | 14.HB.winter | HB | SH | 8196 |
| SH.9 | 2014.11.5 | 14.HB.winter | HB | SH | 7489 |

DF1, core area 1 in Jiangsu Dafeng Milu National Natural Reserve; DF2, core area 2 in Jiangsu Dafeng National Natural Reserve; DF3, core area 3 in Jiangsu Dafeng National Natural Reserve; HB, Hubei Shishou Milu National Natural Reserve.

*DF: our published data ([Wang *et al.* 2019](#_ENREF_1)).

**Table S3** Detailed gut metagenomic information for pandas*

| Sample ID | Clean-reads | Clean-Bases | Average length | Origin | BAC group | Species | Data origin |
| --- | --- | --- | --- | --- | --- | --- | --- |
| 19 | 67,881,149 | 9,867,464,337 | 145.36 | XXL | HP | Giant panda | In this study |
| 20131124-ZX-F03 | 62,349,047 | 9,151,635,679 | 146.78 | ZX | HP | Giant panda | In this study |
| 20140128-zx-F03 | 77,774,061 | 11,537,910,631 | 148.35 | ZX | HP | Giant panda | In this study |
| 23 | 53,538,195 | 7,772,448,526 | 145.18 | XXL | HP | Giant panda | In this study |
| 31 | 53,166,997 | 7,488,933,645 | 140.86 | ZX | HF | Giant panda | In this study |
| 34 | 53,452,895 | 7,766,232,979 | 145.29 | HJ | M | Giant panda | In this study |
| 50 | 53,316,102 | 7,612,446,370 | 142.78 | TT | HF | Giant panda | In this study |
| 9 | 57,047,483 | 8,411,453,987 | 147.45 | HJ | HP | Giant panda | In this study |
| A4 | 60,752,300 | 8,992,034,059 | 148.01 | RP | HF | Red panda | In this study |
| B1-2 | 65,181,154 | 9,677,508,178 | 148.47 | RP | HP | Red panda | In this study |
| B2-4 | 61,454,276 | 9,120,341,274 | 148.41 | RP | HP | Red panda | In this study |
| C14 | 56,064,271 | 8,299,373,106 | 148.03 | RP | HP | Red panda | In this study |
| C18 | 56,456,530 | 8,366,617,158 | 148.20 | RP | HP | Red panda | In this study |
| C2 | 62,648,157 | 9,305,147,273 | 148.53 | RP | HP | Red panda | In this study |
| CDXM18 | 57,156,434 | 8,444,257,007 | 147.74 | XXL | HF | Giant panda | In this study |
| CDXM27 | 56,479,002 | 8,300,348,076 | 146.96 | XXL | HP | Giant panda | In this study |
| CDXM39 | 65,886,310 | 9,753,746,424 | 148.04 | XXL | HP | Giant panda | In this study |
| CDXM40 | 80,837,508 | 11,939,579,426 | 147.70 | XXL | HP | Giant panda | In this study |
| GB1 | 12,117,750 | 1,090,478,845 | 89.99 | QIN | HF | Giant panda | Zhu et al. 2011 |
| GB6 | 24,456,760 | 2,445,393,144 | 99.99 | QIN | HF | Giant panda | Zhu et al. 2011 |
| GB9 | 24,467,016 | 2,446,539,222 | 99.99 | QIN | HF | Giant panda | Zhu et al. 2011 |
| LX3 | 23,696,922 | 2,370,762,117 | 100.05 | LX | HP | Giant panda | In this study |
| LZP1 | 16,688,791 | 1,660,690,975 | 99.51 | XXL | M | Giant panda | In this study |
| TT1 | 6,559,947 | 634,818,332 | 96.77 | TT | HF | Giant panda | In this study |
| TT2 | 44,545,940 | 4,407,262,711 | 98.94 | TT | HP | Giant panda | In this study |

*from our published data ([Zhu *et al.* 2018](#_ENREF_2))

**Table S4** The sample information for 30 Père David’s deer metagenomes*.

| Sample ID | Origin | Collecting time |  |
| --- | --- | --- | --- |
| DF2_15 | DF2 | 09/25/2014 |  |
| DF2_20 | DF2 | 09/25/2014 |  |
| DF2_24 | DF2 | 09/25/2014 |  |
| DF2_25 | DF2 | 09/25/2014 |  |
| DF3_19 | DF3 | 09/26/2014 |  |
| DF3_23 | DF3 | 09/26/2014 |  |
| DF3_28 | DF3 | 09/26/2014 |  |
| DF3_31 | DF3 | 09/26/2014 |  |
| DFW1_15 | DF1 | 11/26/2014 |  |
| DFW1_23 | DF1 | 11/26/2014 |  |
| DFW1_29 | DF1 | 11/26/2014 |  |
| DFW1_34 | DF1 | 11/26/2014 |  |
| DFW2_14 | DF2 | 11/26/2014 |  |
| DFW2_19 | DF2 | 11/26/2014 |  |
| DFW2_26 | DF2 | 11/26/2014 |  |
| DFW2_35 | DF2 | 11/26/2014 |  |
| DFW3_19 | DF3 | 11/27/2014 |  |
| DFW3_27 | DF3 | 11/27/2014 |  |
| DFW3_38 | DF3 | 11/27/2014 |  |
| DFW3_45 | DF3 | 11/27/2014 |  |
| ED1_13 | DF1 | 09/25/2014 |  |
| ED1_17 | DF1 | 09/25/2014 |  |
| ED1_21 | DF1 | 09/25/2014 |  |
| ED1_24 | DF1 | 09/25/2014 |  |
| SH_10 | HB | 11/05/2014 |  |
| SH_16 | HB | 11/05/2014 |  |
| SH_23 | HB | 11/05/2014 |  |
| SH_31 | HB | 11/06/2014 |  |
| SH_45 | HB | 11/06/2014 |  |
| SH_60 | HB | 11/06/2014 |  |

*from our published data ([Zhu *et al.* 2018](#_ENREF_2))

**Figure S1 The variation in predominant gut microbial groups (phylum level) among giant pandas (A, individual HJ; B, individual TT; C, individual LX)**


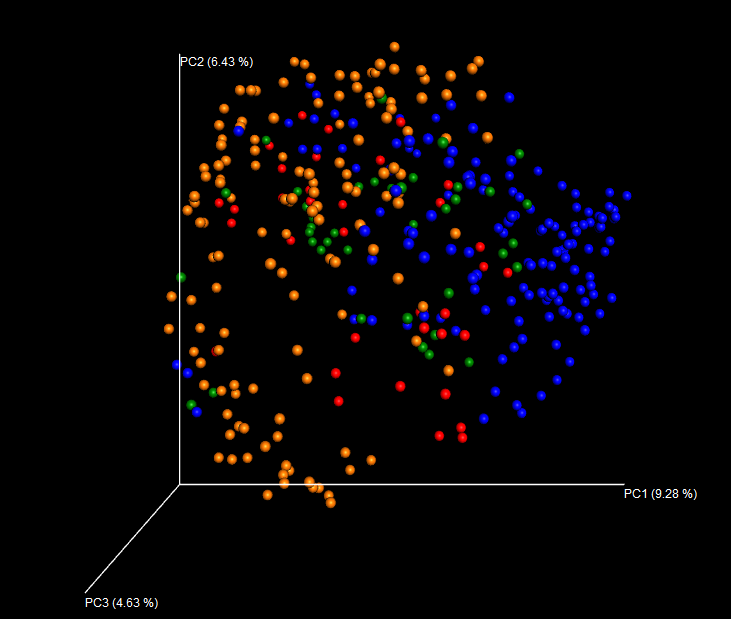


**Figure S2 PCoA analysis using unweighted Unifrac distances for panda gut microbiomes (Red, red panda samples; Blue, translocated panda samples; Green, Xiaoxiangling Mountains samples; Brown, Minshan Mountains samples.)**

**
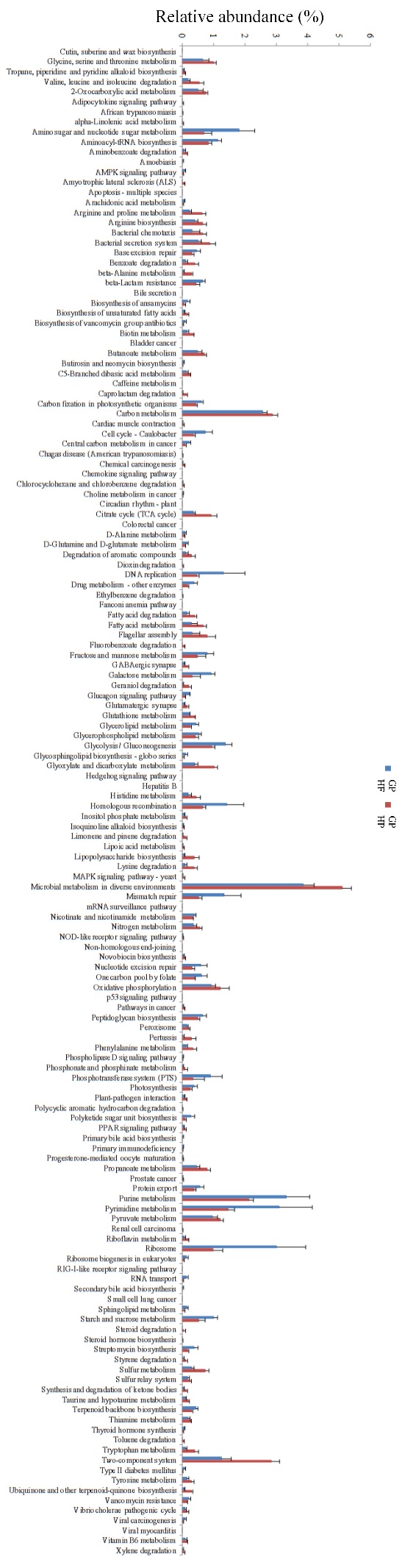
**

**Figure S3 Significantly different relative abundances of ‘KEGG level 3’ pathways between GP-HF and GP-HP (Welch’s *t* test, *p* < 0.05).**

**
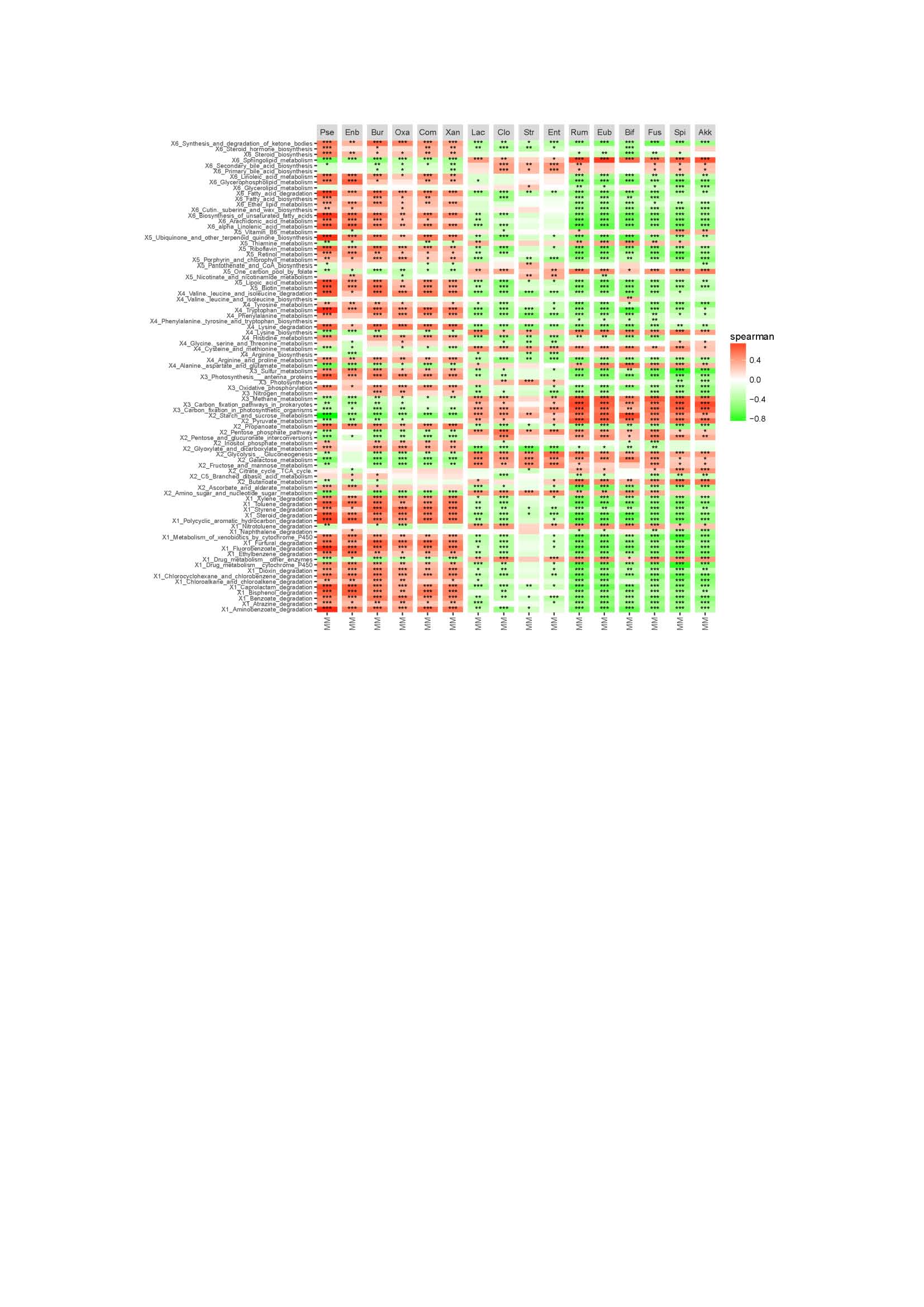
**

**Figure S4. The correlation between enriched pathways and dominant gut bacterial families from 94 gut community metagenomes.** X1, xenobiotics biodegradation and metabolism; X2, carbohydrate metabolism; X3, energy metabolism; X4, amino acid metabolism; X5, metabolism of cofactors and vitamins; X6, lipid metabolism. Pse, Pseudomonadaceae; Enb, Enterobacteriaceae; Bur, Burkholderiaceae; Oxa, Oxalobacteraceae; Com, Comamonadaceae; Xan, Xanthomonadaceae; Lac, Lactobacillaceae; Clo, Clostridiaceae; Str, Streptococcaceae; Ent, Enterococcaceae; Rum, Ruminococcaceae; Eub, Eubacteriaceae; Bif, Bifidobacteriaceae; Fus, Fusobacteriaceae; Spi, Spirochaetaceae; Akk, Akkermansiaceae.

**References**

Wang, L., Ding, J., Yang, Z., Chen, H., Yao, R., Dai, Q., Ding, Y. & Zhu, L. (2019) Père David’s deer gut microbiome changes across captive and translocated populations: Implications for conservation. *Evolutionary Applications,* **12,** 622-635.

Zhu, L., Yang, Z., Yao, R., Xu, L., Chen, H., Gu, X., Wu, T. & Yang, X. (2018) Potential mechanism of detoxification of cyanide compounds by gut microbiomes of bamboo-eating pandas. *MSphere,* **3,** e00229-00218.
